# Structure-Affinity Properties of a High-Affinity Ligand of FKBP12 Studied by Molecular Simulations of a Binding Intermediate

**DOI:** 10.1371/journal.pone.0114610

**Published:** 2014-12-12

**Authors:** Lilian Olivieri, Fabrice Gardebien

**Affiliations:** DSIMB, INSERM, U1134, Paris, F-75015, France, and Université de la Réunion, UMR_S 1134, Faculté des Sciences et Technologies, 15, avenue René Cassin, BP 7151, 97715 Saint Denis Messag Cedex 09, La Réunion, France, and Institut National de la Transfusion Sanguine, F-75015, Paris, France, and Laboratory of Excellence GR-Ex; CNR, Italy

## Abstract

With a view to explaining the structure-affinity properties of the ligands of the protein FKBP12, we characterized a binding intermediate state between this protein and a high-affinity ligand. Indeed, the nature and extent of the intermolecular contacts developed in such a species may play a role on its stability and, hence, on the overall association rate. To find the binding intermediate, a molecular simulation protocol was used to unbind the ligand by gradually decreasing the biasing forces introduced. The intermediate was subsequently refined with 17 independent stochastic boundary molecular dynamics simulations that provide a consistent picture of the intermediate state. In this state, the core region of the ligand remains stable, notably because of the two anchoring oxygen atoms that correspond to recurrent motifs found in all FKBP12 ligand core structures. Besides, the non-core regions participate in numerous transient intermolecular and intramolecular contacts. The dynamic aspect of most of the contacts seems important both for the ligand to retain at least a part of its configurational entropy and for avoiding a trapped state along the binding pathway. Since the transient and anchoring contacts contribute to increasing the stability of the intermediate, as a corollary, the dissociation rate constant 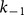 of this intermediate should be decreased, resulting in an increase of the affinity constant 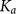. The present results support our previous conclusions and provide a coherent rationale for explaining the prevalence in high-affinity ligands of (i) the two oxygen atoms found in carbonyl or sulfonyl groups of dissimilar core structures and of (ii) symmetric or pseudo-symmetric mobile groups of atoms found as non-core moieties. Another interesting aspect of the intermediate is the distortion of the flexible 80 s loop of the protein, mainly in its tip region, that promotes the accessibility to the bound state.

## Introduction

**Figure 1:**
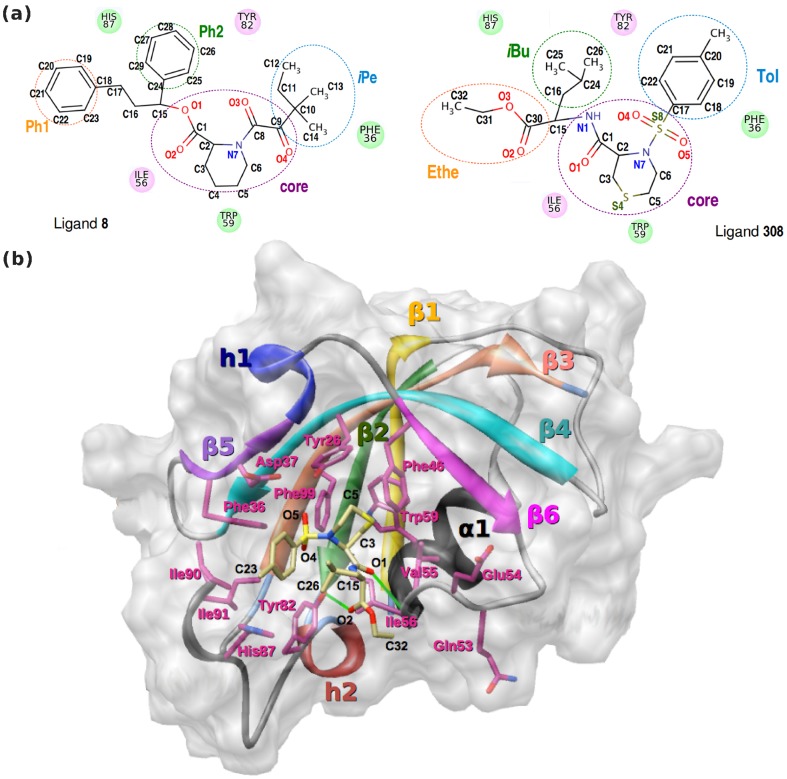
Structures of FKBP12 and of two ligands. (a) Two high-affinity ligands, **8** and **308**, of FKBP12. In the corresponding crystal structures, the orientations of these ligands indicate that Ph1, Ph2, and *i*Pe in **8** are the counterparts of Ethe, *i*Bu, and Tol in **308**, respectively [7], [8]. (b) Experimental structure of the complex between FKBP12 and the high-affinity ligand **308** (compound numbering is from ref [8]). The *β*-sheet and the *α*_1_-helix are highlighted. The two native hydrogen bonds 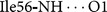 and 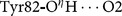 are also represented by thick green lines.

FKBP12 is a 12 kDa enzyme found mainly in the cytosol that catalyzes the peptidylprolyl *cis-trans* isomerization. This protein is a target in the treatment of transplant rejection. For example, the two immunosuppresive exogenous ligands FK506 and rapamycin can bind tightly to FKBP12 with an inhibition constant of 0.6 and 0.3 nM, respectively [1]. These ligands act as dimerization agents between FKBP12 and another protein: with the protein calcineurin, the ternary complex negatively affects cell survival and proliferation [2]; with mTOR, it blocks the T-cell responses by inhibiting lymphokine production [3]. Hyperactive mTOR signaling is linked to tumor growth and its down-regulation by rapamycin or analogues is considered as a promising therapeutic approach for cancer treatment [4]. In neurons, the FK506-bound form of the protein has been associated with neuroprotective properties [5]. Moreover, significant neurite outgrowth are promoted by ligands that bind to FKBP isoforms (all isoforms share a highly conserved FK506 binding domain [6]). Much effort is therefore directed toward finding FK506 analogues with neuroprotective and neurotrophic activities but devoid of the undesirable immunosuppressive activity that is functionally associated with the ligand region responsible for calcineurin inhibition. The two non-immunosuppressive ligands **8** and **308**, shown in Fig. 1, are examples of such high-affinity ligands: the former has an inhibition constant of 10 nM; the latter a dissociation constant in the range 8–14 nM, calculated as 40-fold higher than that of rapamycin [7], [8] (the ligands are labeled as in these reference works). The experimental structure of FKBP12 in complex with **308** is also reported in Fig. 1 [8].

The development of FKBP12 inhibitors represents a major interest for extending the potential of many therapeutic treatments. It is difficult, however, to derive structure-activity relationships for the FKBP12 ligands since highly diverse structures exhibit excellent binding properties. Indeed, the known nanomolar affinity ligands of FKBP12 differ not only by the structure of the central region of the ligand, the core, but also by the peripheral non-core moieties [7], [9]. The core region, as found in the natural products FK506 and rapamycin or in various synthetic derivatives, corresponds to the diketo-pipecolinic acid motif of the ligand **8** shown in Fig. 1a (from the bond C9O4 up to the atom O1). Many derivatives of this core can be found in synthetic compounds, such as in the ligand **308** (from atom N1 up to S8 in Fig. 1a). The non-core moities often make few contacts with the protein in the crystal structure; however, they have a large incidence on the binding affinity. As an illustration, the K*_i_* measured for the ligand **8** and its close analogue **5** [7], which is obtained by the removal of the aromatic ring Ph2 in **8**, are 10 and 110 nM, respectively. Though no crystal is available for **5**, a free energy perturbation study has shown that its binding mode is similar to that of **8** [10]. In the crystallographic structure of FKBP12–**8**, the Ph2 ring is protruding into the solvent and does not make contacts with the protein. Therefore, in their respective fixed complexes, the two ligands **5** and **8** make the same number of contacts with the protein and they are also expected to have roughly the same buried surface. By considering these similarities between fixed **8**- and **5**-bound complexes, the use of empirical enthalpic terms for the van der Waals contacts, electrostatic interactions, and hydrogen bonds combined with a hydrophobic contribution (often taken as proportional to the buried surface area) would not account for the ten-fold difference in affinity. Holt *et al.* [7] have attributed this difference mainly to an intramolecular contact Ph2 makes in **8**, which would not satisfactorily explain the large binding free energy difference. To unravel the factors responsible for the binding free energy difference of various FKBP12 ligands, the ligand dynamics was also examined by undertaking molecular simulations [10]–[12]. Wang *et al.* [11] have reproduced accurately the experimental binding free energy of FKBP12 and a series of analogues of **8**. Their study underlies the fact that the binding free energy is dominated by the contribution of hydrophobic contacts but also by the entropic penalty due to the loss of translational, rotational, and conformational degrees of freedom of the ligands. Actually, the hydrophobic contacts seem not to be a primary determinant of the ligand affinity, as demonstrated by the experimental work of Yang *et al.* [9] on remote analogues of ligand **8**. In this study, an engineered hole has been created inside the protein binding pocket to fit various hydrophobic moieties of the FKBP12 ligands with the aim of enhancing their binding properties. Unexpectedly, the binding affinity of these ligands are unrelated to the extent of complementarity between the protein and its ligand, *i.e.* to the number of hydrophobic contacts. The above theoretical and experimental results thus reveal that the ligand entropy contributes significantly to the binding free energy, which proves to be most awkward in attempts at scoring fixed FKBP12-ligand complexes or at deriving structure-affinity relationships of the ligands.

In this work, we characterized an intermediate state, IS308, for the unbinding pathway of the complex between FKBP12 and the high-affinity ligand **308**, which displays a rather different core structure as compared to **8** (Fig. 1). This work shares the similar objective of our previous work on the complex FKBP12-**8** [13]. By relying on the structural characteristics of the intermediate, our objective was to delineate the essential structural features of the ligand **308**, such as the role played by its core and non-core regions, that can account for its high affinity. Since the ligand **8** and **308** are structurally dissimilar, we also wanted to challenge our previous results and conclusions.

As we shall see, the nascent intermolecular contacts in this intermediate could play a major role in the affinity of the ligand. Generally speaking, for the complexation between a protein P and its ligand L, the association constant 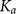, which is a measure of the affinity, can also be written as the ratio between 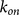 and 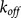, the association rates of association and dissociation, respectively:
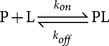
(1)

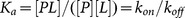
(2)

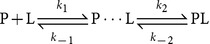
(3)

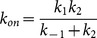
(4)

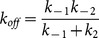
(5)


It is expected that the type and extent of intermolecular interactions developed in the binding intermediate P
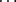
L will have an incidence on its stability and, hence, on its dissociation rate 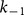. The relationship between the stability of the intermediate and the affinity was shown by Mittag *et al.* for the binding of two phosphopeptide ligands to the N-terminal SH2 domain of PI3-K [14]. In this study, the authors have found that the longer is the lifetime of the intermediate, the higher is the affinity of the ligand. We can hypothesize that, in their case, the rate of dissociation 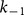 of the intermediate decreases as the lifetime of the intermediate increases, thereby resulting in an increase of the affinity according to equations 5 and 2. The authors have also stressed that such intermediates may be more common in protein-ligand systems than previously anticipated.

In our approach, we aim at characterizing the nature and the extent of the contacts formed by the ligand **308** in the binding intermediate, as these contacts may have an incidence on the lifetime of this state, and thus on the dissociation rate 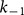. This analysis performed on a binding intermediate for this ligand and the comparison with the results previously obtained for the structurally dissimilar ligand **8** will also provide clues regarding the recurrent structural motifs that are determining for high binding properties of FKBP12 ligands. The same protocol we used in our previous study of the system FKBP12-**8** was followed to search for a binding intermediate state and will be briefly described in the following section. All the results for the binding intermediate are presented and discussed in the sections 3 and 4, respectively. These results provide a consistent picture of the binding intermediate with a well-defined position of the ligand that forms very few permanent and numerous transient contacts with the protein.

## Materials and Methods

### Preparation of the system

The X-ray coordinates of the complex between the protein FKBP12 and the synthetic inhibitor **308** (PDB code: 1J4I [8]; Fig. 1) were used for the simulations of the bound state. The all-atom Charmm22 force field [15] was used for the protein and the ligand; however, a few parameters for the ligand were optimized by a fitting procedure [16]. In particular, we derived the charges on the atoms of the core region and we determined the torsional parameters for the rotation around the bonds C31–O3, C30–C15, C15–N1, C1–C2, C3–S4, N7–S8, and C17–S8. Much effort has been directed toward obtaining realistic barrier heights for each of these seven torsion angles because of the steric constraints that exist in the core region due, in particular, to the amide bond that occupies an axial position of the central ring [17]. The dihedral potentials were optimized based on energy scans computed with the program Gaussian03 [18] and GAMESS-US [19] at the HF/6-31G* level. To improve the accuracy of the torsion barriers, the fitting was performed with the energies of the minima and maxima of the potential energy surface that were refined at the MP2/6-31G*//HF/6-31G* level.

In the crystal structure of the complex, the ethyl part C31-C32 of the ligand is missing. We first generated guess positions for these atoms and added the hydrogen atoms using Chimera tools [20]. Using a regular dihedral interval, nine new positions of the ethyl part were then generated by rotation about the bond C31–O3. By fixing all the atoms of the protein and of the ligand but those of the ethyl part, the nine structures generated were subsequently minimized and subjected to MD simulations at 300 K (two production runs of 100 ps per initial structure). Finally, the 18 final positions generated only for the ethyl group were all minimized and the structure with the lowest energy was selected.

### Simulations of the bound state

The same protocol as in our previous study was used and is hereafter summarized [13]. All the molecular dynamics simulations that used an implicit or explicit solvation model were performed with the CHARMM program [21] using the leap-frog integrator and a time step of 1 ps. SHAKE [22] restraints were applied to the bonds containing hydrogen atoms. Langevin dynamics (LD) [23], with a friction coefficient chosen at 60 ps^−1^, were used to simulate the bound state. The Effective Energy Function 1 (EEF1) [24] was chosen to compute the solvation free energy of the atoms in the LD simulations of both the bound state and the unbinding process.

For the LD simulations, the system was initially energy-minimized and then heated from 0 to 300 K in steps of 25 K (for 600 ps); different random seeds for the distribution of initial velocities led to six independent simulations. The heating run was followed by an equilibration divided in two periods of 500 ps each. For the heating and for the first equilibration periods, the sets C1, C2, C3, and C4 of NOE restraints were used (the sets of CHARMM NOE restraints are defined in the Supporting Information and are derived from the crystal structure). After the second equilibration period that used the sets C1, C2, and C3 of NOE restraints, the six LD production runs were conducted with the same sets C1, C2, and C3 of NOE restraints for a total simulation time of 53 ns (S1 Table). The sets C1 and C2 of NOE restraints were used to prevent the distortion of the 80s loop; the set C3 to mimic the presence of two water molecules [25], [26]; the set C4 to reduce the fluctuations of the residues involved, to avoid the distortion of the 80s loop, and to achieve agreement with the intermolecular distances measured in the crystal. The Supporting Information provides the definition of the sets C1 and C2 (S1 Text) as well as those of the sets C3 and C4 (S2 and S3 Tables, respectively).

To compare the results obtained with the LD simulations, the bound state was also simulated by using six independent stochastic boundary molecular dynamics (SBD) simulations [27] that included a sphere of water molecules with a diameter of 25 Å centered on the complex and a buffer region of 3 Å. Water molecules that had an oxygen atom within 2.8 Å of the protein or ligand heavy atoms were deleted. The nonbonded interactions were cut off at 14 Å with a force switching function used between 11 and 14 Å. The hydrated form of the complex was then energy-minimized in four steps. First, only the water molecules were subjected to 1000 steps of steepest descent energy minimization (gradient tolerance of 1 kcal/mol/Å) while the protein and ligand atoms were kept fixed. Second, the ligand and the solvent were harmonically restrained while the protein was energy-minimized with 1000 steps of steepest descent (gradient tolerance of 0.5 kcal/mol/Å). Third, only the harmonical restraints on the solvent were applied while the ligand and the protein were energy-minimized with 1000 steps of steepest descent (gradient tolerance of 0.1 kcal/mol/Å). Fourth, all the atoms of the system were subjected to 5000 steps of steepest descent energy minimization (gradient tolerance of 1 kcal/mol/Å).

For the SBD simulations, the system was then heated from 0 to 300 K using a step of 10 K by assigning velocities every picosecond (for 30 ps) and using three different random seeds. During the heating, the sets C1, C2, C4, and C5 of NOE restraints were used. The heating run was followed by two successive equilibration runs of 300 and 150 ps, with the atomic velocities rescaled. The sets C1, C2, C4, and C5 of NOE restraints were applied during the first equilibration run; however, all the restraints were removed during both the second equilibration and the production runs. The sets C1, C2, and C4 were used for the same reasons as for the LD simulations; the set C5 was used to achieve agreement with the experimental position of the ligand in the binding pocket (its definition is provided in S1 Text; see also S1 Table).

Prior to the unbinding simulations, we checked that the LD simulations of the bound state provided results in agreement with both the X-ray structure and the SBD simulations. The purpose in using LD simulations for the description of the bound state is twofold: (i) to validate the force field and solvation parameters that are also used in the subsequent step of unbinding simulations, and (ii) to obtain initial structures for the unbinding simulations. Implicit solvent simulations are required for the unbinding step as has been recommended for unfolding or unbinding events [28].

### Unbinding simulations and refinement protocol

The same unbinding and refinement protocols as in our previous work were followed [13]. Fifty-seven structures were extracted from the six LD simulations of the bound state (taken as the last structure of a simulation run or at a 1-ns interval within a simulation) and were used to unbind the ligand from the protein.

As the unbinding is a long-extended process [29], a perturbative force was added to the molecular potential energy function for each unbinding simulation. The time-dependent perturbation implemented in the Biased Molecular Dynamics (BMD) [28] method was used. It is noteworthy that, among the perturbation methods that have been recently compared, BMD is the method that causes the least perturbation in a system [30]. In this method, a quadratic time-dependent perturbation is introduced in the system only when the distance between the ligand atom N7 and the C*_α_* atom of Glu5 decreases (d*_RC_*); otherwise, no external perturbation is applied, and the increased distance d*_RC_* is taken as the new reaction coordinate for the next time step. The BMD method allows sampling the unbinding pathway under quasi-equilibrium conditions for an implicit solvation model, as previously shown [28]. Implicit solvation model such as LD is required since when large conformational changes are forced to occur in a short time compared to the experiment, the relaxation of an explicit solvent is too slow and add artifacts. Furthermore, in a comparison study of the three most commonly used forced unbinding simulations method (targeted, steered, and biased molecular dynamics), Huang *et al.* have shown that the BMD simulations cause the least perturbation in a system [30].

**Figure 2:**
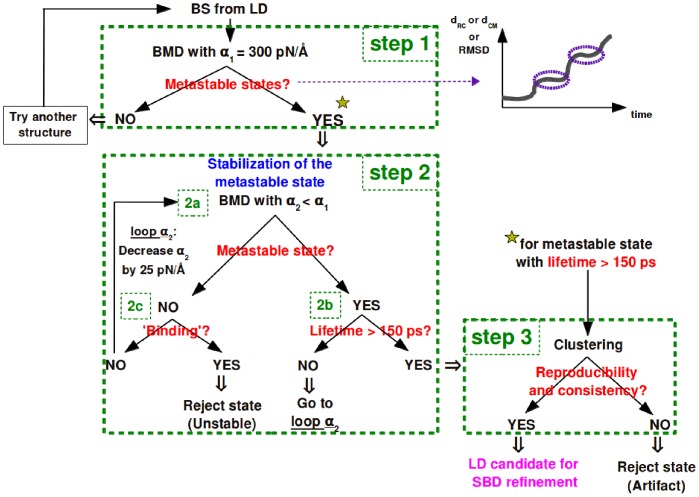
Protocol to find the LD candidates for the binding intermediate. Step 1: unbinding simulations on each of the 57 structures separated by 1 ns and selected from the six LD simulations of the bound state. The star above ‘YES’ indicates that the metastable states with lifetime greater than 150 ps are also added to the set of simulations used in the clustering step (step 3). Step 2: In 2a, for each metastable state from step 1, BMD simulations are performed with *α*_2_ lower than *α*_1_ to extend the lifetime of the metastable state found in step 1. In 2b and 2c, metastable states with lifetime > 150 ps are added to the pool used in step 3; otherwise, a lower *α*_2_ is used for restarting the simulations. ‘Binding’ checks wether the ligand returns to its position in the bound state. Step 3: The metastable states with lifetime greater than 150 ps are clustered into subsets that exhibit related structural features (see text). Reproducibility and consistency within a cluster are critical in finding the LD candidates for further refinement.

In the initial BMD simulations (step 1 in Fig. 2), the constant of the perturbative force (parameter 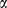) was chosen at 300 pN/Å, since this value enabled the unbinding of the ligand within a time limit of 5 ns. The unbinding process was followed by monitoring the time evolutions of d*_RC_* and of d*_CM_*; the latter represents the distance between the center of mass of the binding pocket and each of the four centers of mass of the four ligand moieties (core, *i*Bu, Tol, and Ethe). This procedure was also followed by Curcio *et al.* in their study of forced unbinding of fluorescein from anti-fluorescein antibody FITC-E2 [31]. We also monitored the root mean square deviations (RMSDs) for both the protein and the ligand moieties (core, *i*Bu, Tol, and Ethe) with the C*_α_* atoms aligned on those of the X-ray structure of the bound state.

**Figure 3:**
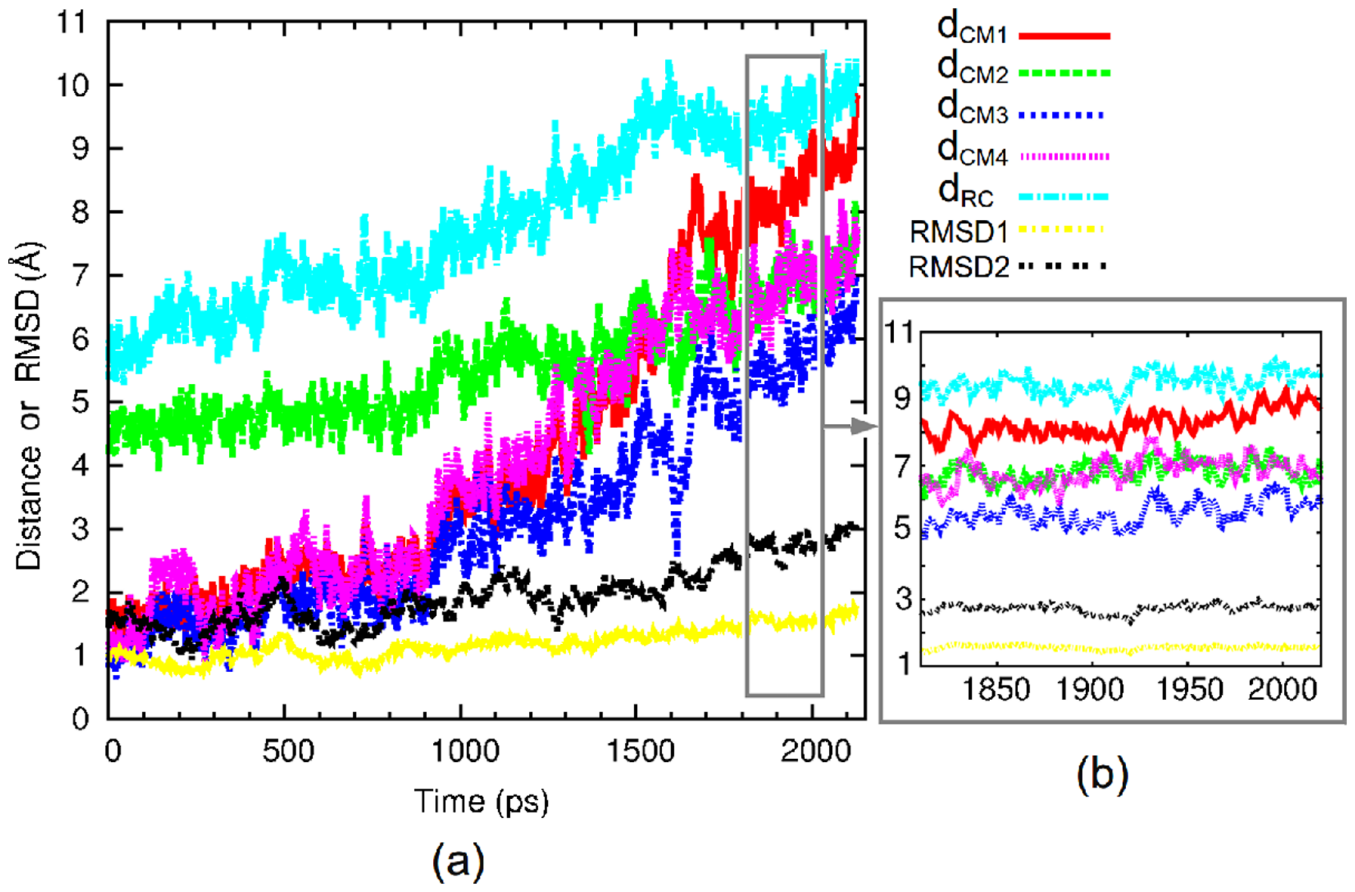
Time evolutions of d*_RC_*, d*_CM_*, and RMSDs calculated for the protein. d*_CM_*_1_ to d*_CM_*_4_ represent the distances between the center of mass of the binding pocket and the center of mass of the moieties core, Ethe, *i*Bu, and Tol, respectively. The values of RMSD1 and RSMD2 were calculated for the protein after alignment A1 and for the 80 s loop after alignment A2, respectively (see text for the definition of these alignments). (a) Time evolutions from the native complex; (b) Specific focus on a particular interval where all these quantities remain stable.

From the unbinding simulations (step 1 in Fig. 2), many metastable states were found by relying on the time evolutions of d*_RC_*, d*_CM_*, and RMSDs; constant mean values for these distances and RMSDs for periods of hundreds of picoseconds constitute useful indicators. Fig. 3 illustrates the time evolution of all these distances and RMSDs for an example of metastable state of about 200 ps. The metastable states from step 1 with lifetime greater than 150 ps were used both in stage 2a and in the clustering step of the protocol (step 3; *vide infra*). However, most of the metastable states found in step 1 have a lifetime that is lower than 150 ps. In this case, the last structure obtained in the portion of the simulations that corresponds to constant mean values of d*_RC_*, d*_CM_*, and RMSDs was chosen for the step 2 whose purpose is to increase as much as possible the lifetime of these metastable states. Indeed, during the unbinding in step 1, the intermolecular distances was increased and the number of intermolecular contacts was decreased, thereby reducing the intermolecular forces. Hence, in step 2, since a smaller value of the perturbative force is needed, the simulations were started by gradually decreasing the value of 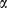 by steps of 25 pN/Å in the range 50–250 pN/Å to further stabilize the metastable states. More simulations with stable values of d*_RC_*, d*_CM_*, and RMSDs were then obtained in the step 2b. If the lifetime of such a metastable state is higher than 150 ps, then it is used in the clustering step 3. If not, the simulations describe a metastable state that is too short and, to increase its lifetime, the last structure in the portion of the simulations that corresponds to constant mean values of d*_RC_*, d*_CM_*, and RMSDs was used as starting structure in the step 2a with a lower value of 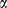. In the step 2c, no metastable state was found because 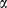 was either too low, leading to the binding of the ligand (the simulations are discarded), or too high, leading to a fast unbinding process. In the latter case, the same simulation is restarted in the step 2a but by using a value of 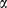 that is decreased by 25 pN/Å to reduce the perturbative force and to slow the unbinding process. By slowing the unbinding process, one could increase the sampling of the potential energy surface for the ligand coordinates in the vicinity of the binding pocket and, thus, possibly obtain more metastable states that describe an intermediate binding state. Simulations with longer lifetime were indeed obtained that exhibited stable values of d*_RC_*, d*_CM_*, and RMSDs for the protein and for the ligand. The simulations with a lifetime greater than 150 ps were added to the pool of states analyzed in the step 3.

In step 3 of Fig. 2, all the metastable states with a lifetime greater than 150 ps, obtained from both the unbinding (step 1 in Fig. 2) and the simulations performed to increase the sampling (step 2 in Fig. 2), were clustered into subsets. The following four criteria were used to cluster and to select the LD simulations that could model the binding intermediate. Only one subset of LD simulations passed these criteria; S4 and S5 Tables give the various distances and RMSDs that were calculated for this subset and were used to cluster the simulations in this step 3. First, the trajectories must be stable for periods of at least hundreds of picoseconds. Within a subset of metastable states, the values of d*_RC_*, d*_CM_*, and RMSDs for the protein and for the ligand must remain constant during the course of each simulation as well as consistent among all the simulations. The same structural criterion was followed by Li and Daggett in the search of an intermediate state along the unfolding pathway of barnase [32]. Second, the structure of the protein must not be significantly altered. Third, the set of LD simulations must sample a range of conformations that are structurally related and, in particular, the positions of the ligand with respect to the protein must form a tight cluster. Central to this last criterion is the reproducibility and consistency that constitute useful indicators that the results have sufficiently converged so as to provide a meaningful average picture of the intermediate. Fourth, as we hypothesize that the intermediate state is an obligatory step from the freely diffusing molecules toward the bound state, we also checked that the intermediate was structurally close to the bound state in relative separation and relative orientation between the two molecular partners. For the selected subset of LD simulations, the time average values of the d*_CM_* distances reveal a stable position of the ligand in the individual simulation (all the fluctuations are below 1 Å and are even less for the core region of the ligand, 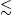 0.5 Å; S4 Table). The ensemble average values of d*_CM_* reveal a consistent position of the ligand throughout all of the simulations (standard deviations lower than 0.68 Å; S4 Table). The RMSDs in S5 Table are large since they measure the shift in the position of the four ligand moieties in IS308 relative to their positions in the native complex. Most important is the fact that these values exhibit fluctuations that are lower than 1.2 Å within all of the simulations, and taken together, they indicate an overall consistent position of the ligand within all the set of simulations (standard deviations lower than 0.56 Å; S5 Table). In contrast, in the other sets of simulations, though the fluctuations for the intermolecular distances and RMSDs are less than 1 Å within each simulation, the standard deviations are calculated to be above 2 Å for a given set of simulations.

This subset of LD simulations was selected for the refinement procedure that used independent SBD production runs with no biasing forces or restraints on the system. The purpose in using an explicit solvent representation to further characterize the intermediate state is twofold: (i) to further probe the stability of the binding intermediate located using LD simulations and (ii) to refine the results since explicit solvent model is known to provide a more realistic description of a system. For the SBD simulations, the same protocol as for the bound state was used, except that the water molecules were here added to the selected structures in a sphere of 22 Å in diameter centered on the binding site. This refinement protocol was performed by 17 independent SBD simulations with no biasing forces or restraints on the system for a total run of 25 ns of production [13]. All the data that are reported for the bound state CS308 and for the binding intermediate IS308 correspond to ensemble averages.

## Results

**Figure 4:**
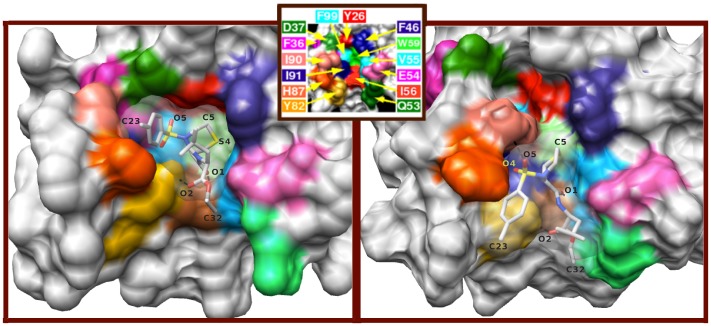
Comparative analysis of the position of the ligand in the binding pocket of FKBP12 in the crystal (left) and in the binding intermediate IS308 (right). The protein residues are color-coded according to the inset at the top of the figure. The two contacts 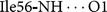 and 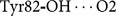 are represented by dashed lines, along with the contact 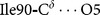 that is shown only for IS308.

In this section, the results for the intermediate IS308 are presented. A snapshot of IS308 is shown in Fig. 4 along with the crystal structure for purposes of comparison. We shall analyze the structure of the protein, the position of the ligand in the binding site, and the intermolecular contacts between FKBP12 and the ligand.

### Structure of the protein

The structure of the protein in the intermediate state IS308 was analyzed by performing two types of RMSD calculations with the C*^α^* atoms aligned on those of the X-ray structure of the bound state. In the first type, hereafter named A1, all the C*^α^* atoms are aligned on their reference position in the crystal. In the second, named A2, all the C*^α^* atoms are aligned on their reference position except those of a given group of atoms G; this allows examining the displacement of the group G with respect to the rest of the protein. The table 1 gives the values of RMSD. For IS308, the average RMSD calculated by A1 for the C*^α^* atoms of all the residues, except those that are in the 80s loop, is 1.11 Å. On the other hand, for the 80s loop, the value calculated by A2 indicates a global distortion of 4.01 Å in IS308 and of 2.15 Å in the SBD simulations of the complexed state CS308, the latter value being similar to the average value calculated for all the C*^α^* atoms in CS308 (1.9 Å, not shown in table). For IS308, a detailed analysis of the various sub-regions in the 80s loop indicates that the segment Ala84-Pro93 undergoes a large displacement and contributes mainly to the distortion of this loop (RMSD of 4.57 Å obtained by A2); in particular, the largest displacement inside this residue range is found for the tip of the loop, Gly89-Ile90 (5.7 Å; not shown in table). This feature is similar to what was found in the binding intermediate for the ligand **8**, IS8 (4.5 Å for the residue range 88–93 and a displacement of 5.5 Å for Gly89) [13]. Note also that this loop is rich in proline and glycine; it contains three of each of these residues. This result is in agreement with other experimental observations on protein-ligand complexes: the presence of glycine residues close to the site of complexation has been described as crucial for the recognition of ligands by favoring the local fluctuations or changes in loop structure that contribute to the incoming of the ligand [33]–[35]. More specifically, for the protein FKBP12, previous studies have demonstrated (i) the distortion of the 80s loop structure around Gly89 and (ii) the importance of this loop for the recognition of the ligands [36], [37]. Moreover, by comparing FKBP12 in its bound and unbound conformations, Ivery and Weiler have deduced that the 80s loop must undergo a movement during the complexation with the ligand FK506 [38]. The same conclusion was drawn by Wilson et al. after comparing the X-ray structures of unbound, FK506-, and rapamycin-bound forms of FKBP12 [39]. In line with these results, in this intermediate state IS308, as in our previous study on IS8, we found consistent results showing that the 80s loop is distorted mainly in the tip region.

**Table 1:** RMSDs (Å) of the C*^α^* atoms from the respective X-ray structural positions.

	nonloop^a^ (A1)	80 s loop (A2)	Tyr82-Gly83 (A2)	Ala84-Pro93 (A2)	His94-Ala95 (A2)
CS308	1.76 ± 0.12	2.15 ± 0.29	1.12 0.33	2.45 0.19	0.95 0.17
IS308	1.11 0.05	4.01 0.28	1.34 0.18	4.57 0.25	1.73 0.16

For all the RMSD calculations, the alignments A1 and A2 were based upon the protein C*^α^* atoms (the group of atoms G for A2 corresponds to the 80 s loop or to its three sub-regions 82–83, 84–93, and 94–95; see text for the definition of A1 and A2). For comparison, their counterparts calculated for the complexed state CS308 are also given.

a All C*_α_* atoms but those of segment 82–95.

### Position of the ligand in the binding site of FKBP12

The position of the ligand was analyzed in terms of the distances, 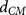, between the center of mass of the protein binding pocket and each of the four centers of mass of the four ligand moieties (core, *i*Bu, Tol, and Ethe); we checked that they remained constant during the course of each simulation as well as consistent among the set of simulations. To measure the deviations from the X-ray structure, RMSD calculations for the heavy atoms of each ligand moiety were also performed with the alignment A1. The average values of 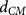 and RMSD obtained for IS308 and CS308 are given in table 2. In the individual simulations of IS308, the values of 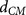 and RMSD calculated for the core moiety reveal a stable position of the ligand core since the fluctuations of these two quantities remain in the range 0.3–0.5 Å for the core (not shown in table). Moreover, the low standard deviations of these quantities (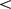 0.2 Å) in the table 2 indicate that the positions of the ligand with respect to the protein are not spread sparsely but rather form a tight cluster. The standard deviations of 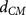 and RMSD for the non-core moieties are lower than 0.4 Å, also indicating overall consistent values throughout all the simulations. For IS308, the analysis of the average values of RMSD of the four ligand moieties reveals that the moieties that are closest and farthest from their respective native positions are Ethe and Tol, respectively (for IS8, the same conclusion was drawn for the two counterparts Ph1 and *i*Pe in **8**). In particular, the Tol moiety in **308** and its counterpart *i*Pe in **8** both interact with the mobile side chain of His87, which is at the entrance of the binding pocket, to form many transient contacts (*vide infra*). Another similarity between these intermediates concerns the oxygen atoms O4 in IS308 and O3 in IS8 that both points to the small side cavity defined by the residues 82, 87, 90, and 91 of the distorted 80s loop (Fig. 4).

**Table 2:** Average distances (Å) between the center of mass of the core and that of the binding pocket (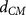), and RMSDs (of the heavy atoms only, in Å) of the ligand and of its four moieties from the respective X-ray structural positions.

	d 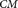			RMSD	
	Exp.	CS308	IS308	CS308	IS308
ligand	3.24	3.15 0.12	7.47 0.17	1.42 0.21	6.62 0.24
Ethe	6.07	6.09 0.09	8.71 0.31	1.18 0.12	4.57 0.32
*i*Bu	5.16	5.79 0.23	9.28 0.19	2.38 0.38	7.61 0.28
core	1.34	1.24 0.10	5.15 0.14	0.51 0.04	5.96 0.17
Tol	4.50	4.80 0.06	8.88 0.27	1.53 0.42	8.30 0.38

For all the RMSD calculations, the alignment was based upon all the protein C*_α_* atoms. Exp. corresponds to the X-ray structure.

In the next section, we will analyze the permanent and transient interactions that can account for the reported stability of the ligand in IS308.

### Analysis of the interactions in IS308

For purposes of comparison, the contacts between the ligand and FKBP12 were analyzed by using the same criteria as in our previous work [13]. In particular, we restricted the numbers of contact distances to the atom pairs separated by less than 4 Å in more than half of, at least, one simulation. In S6 Table, the time average and ensemble average values are reported for 71 contact distances that satisfied these criteria. Among all these distances, only seven contacts correspond to persistent interactions that are observed in all the frames and with standard deviations lower than 0.25 Å; these permanent contacts can be classified into five groups: Glu54-O
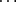
{C15, C16}, Val55-{C, C*^α^*}
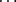
O1, Ile56-N
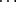
O1, Tyr82-O^*η*^···C2, and Ile90-C^δ^···O5. Among these permanent contacts that involve the atoms O1 and O5, it is noteworthy that they all have their counterparts in the intermediate IS8: Ile56-N/Val55-{C, C*^α^*}
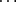
O1 equivalent to those formed by the atom O2 in IS8; Ile90-C^*δ*^···O5 equivalent to Ile90-C^*γ*2^···O3 in IS8 [13]. Thus, these results indicate that the permanent contacts in IS8 and in IS308 are similar, despite the dissimilar structures of these ligands. Moreover, in these two intermediates, the two oxygen atoms O2 and O3 in **8** or O1 and O5 in **308** act as two anchoring points for the ligand at the entrance of the binding pocket, restraining overall translational and rotational mobilities; this allows the peripheral moieties to fine-tune their interactions with the protein in this binding step. This result, along with the preceding RMSD and distance analysis, indicates that the overall orientation of these ligands is similar in these intermediate states. We shall see other similar features between these two intermediates.

In IS308, it must be emphasized that none of the hydrogen bonds Ile56-N–H
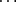
O1 and Tyr82-O^*η*^–H
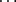
O2 are formed. For these two bonds, the average distances between O1 and O2 and the hydrogen atom are 2.78 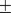 0.34 Å and 2.52 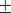 1.13 Å, and the average hydrogen bond angles are 123 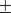 12° and 140 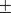 40°, respectively. Hence, Ile56-N
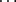
O1 should be viewed as a permanent van der Waals contact and Tyr82-O^*η*^···O2 as a transient van der Waals contact. This result is also similar in IS8: **8**-O2 makes a van der Waals contact with Ile56 and **8**-O3 a transient contact with Tyr82 [13]. Since the two hydrogen bonds are not yet formed in IS8 or IS308 with the residues Ile56 and Tyr82, their formation would thus represent a major enthalpic driving force in going from the intermediate state to the complexed state.

**Figure 5:**
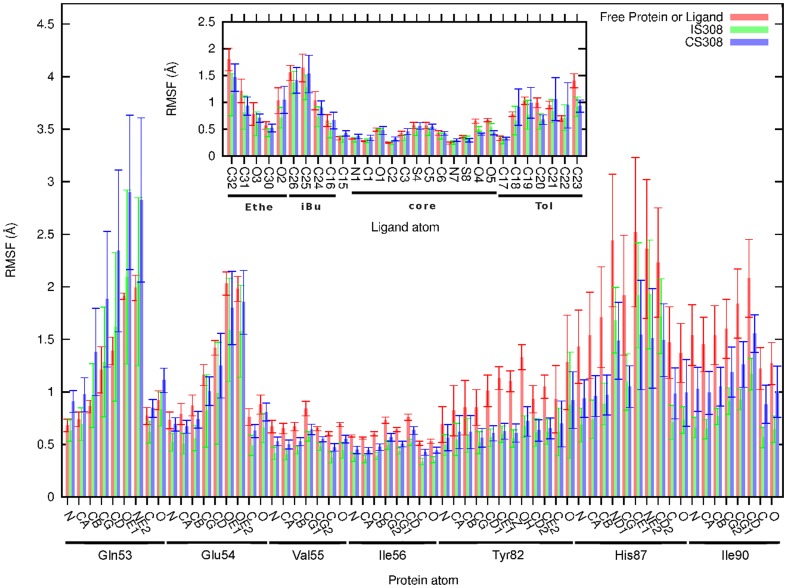
RMSFs derived for the binding intermediate. RMSFs calculated for the main residues 53–56, 82, 87, and 90 that are in contact with the ligand in IS308; the corresponding values for the bound state (CS308) are also reported for comparison. The RMSFs for the ligand atoms in IS308 and the bound state are shown in the inset. For purposes of comparison, the RMSFs of these seven residues in the free protein and those of the free ligand (inset) were also calculated by averaging over four 2-ns and eight 250-ps SBD simulations, respectively.

The RMSFs of the atoms of the protein residues that are involved in most of the contacts with the ligand were calculated for IS308, for CS308, and for the unbound protein (Fig. 5). Those of the ligand in IS308, CS308, and the free state were also calculated and are reported in the inset of Fig. 5. The comparison of the RMSFs of the ligand between the three states indicates that the largest differences are 0.5 Å, calculated for the extremities C23 and C32 between the free state and IS308. The RMSFs of the peripheral moieties *i*Bu, Tol, and Ethe in IS308 are slightly lower than in CS308; these small differences (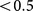) should be ascribed to the numerous transient intra- and intermolecular contacts monitored for these moieties in IS308, as seen below, and that moderately decrease their mobility.

**Figure 6:**
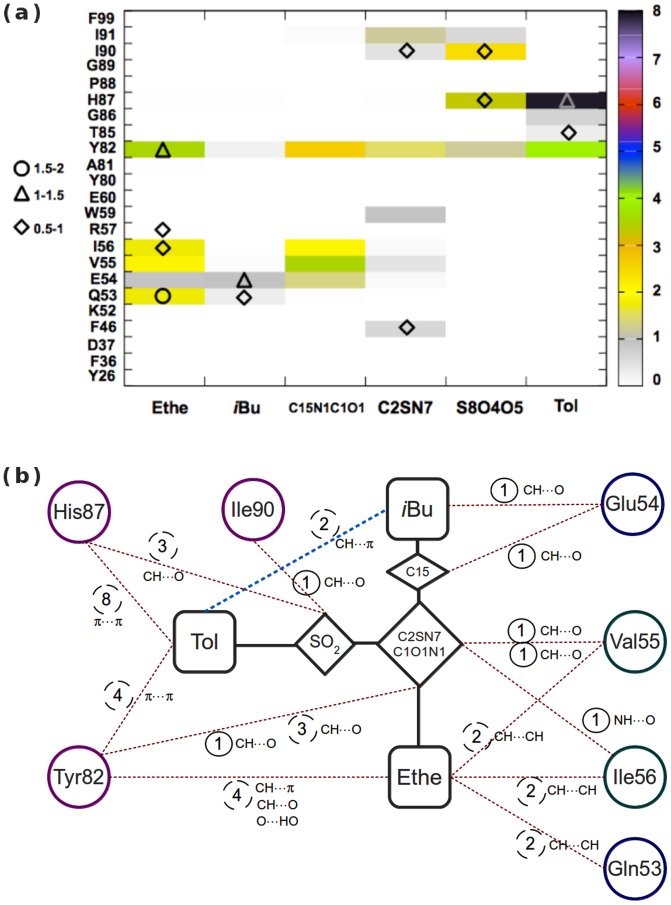
Analysis of the intermolecular contacts in the binding intermediate. (a) Average number of contacts between each pair of interacting groups of atoms that consists of a ligand moiety and a protein residue (color scheme on the right), and average number of water molecules in contact with both a ligand moiety and a protein residue (diamond, triangle, and circle on the left are for a number of bridging water molecules in the intervals]0.5; 1],]1; 1.5], and]1.5; 2], respectively). The core region of the ligand is further subdivided into the central C2SN7 ring and the S8O4O5 and C15N1C1O1 groups of atoms. All types of contacts (cutoff of 4 Å) were first obtained for each SBD simulation; the average over the 17 independent simulations was then derived. (b) Schematic representation of the main protein-ligand contacts. The number of permanent and transient contacts are circled in solid and dashed lines, respectively; the type of non-covalent interaction is indicated in each case. The transient contacts with an average count ≤ 1 are not shown; the following are with an average of one: 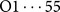, 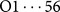, 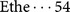, 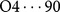, and 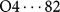. The ligand intramolecular contact is shown as a dashed blue line. Positions and distances for all moieties are not realistically represented.

In order to quantify all the transient interactions between protein and ligand groups of atoms, we determined, by using a 4-Å cutoff, the contacts between each pair of interacting groups of atoms that consists of a ligand moiety and a residue. The average total counts are shown as a contact map in Fig. 6a. From these average total counts, the number of transient interactions formed between a ligand moiety and a residue of FKBP12 can then be obtained by subtracting the related permanent interactions (if any and as defined in the beginning of this subsection). A schematic representation of the main interactions are also given in Fig. 6b where the seven permanent interactions, mainly of type CH
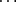
O, with the residues 54, 55, 56, 82, and 90 are reported. We can thus see that all the ligand moieties are involved in numerous transient contacts; however, since their lifetime is, on average, on the order of tens of picoseconds, this results in average interatomic distances that are, most often, above 4 Å and in standard deviations above 0.5 Å (S6 Table).

**Figure 7:**
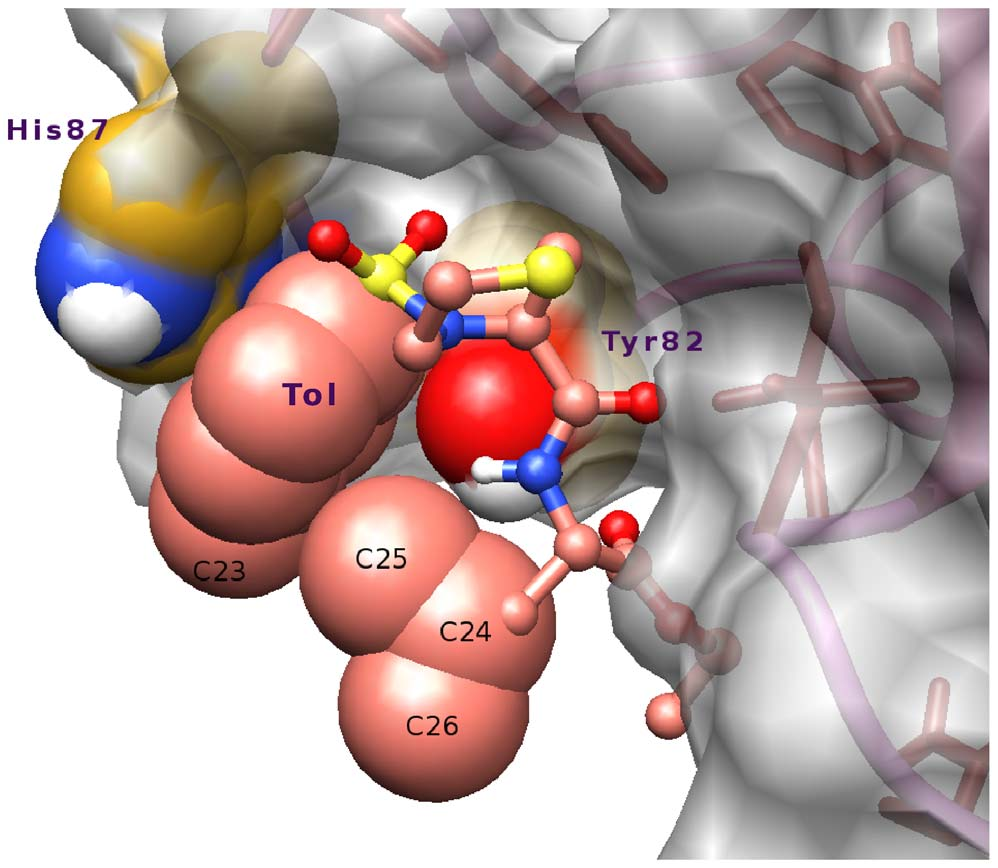
Ligand 308 in the pocket of the binding intermediate. Snapshot showing the Tol moiety surrounded by the atom C25 (or C26 after dihedral rotation) of the ligand as well as by the side chains of His87 and Tyr82, forming numerous transient contacts (the carbon atoms of the ligand are in salmon).

Besides the permanent contacts that the atoms O1 and O5 of the core moiety make, these two atoms also participate in transient contacts: O1 makes one transient CH
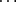
O contact with each of the residue Val55, Ile56, and Tyr82 (for clarity, only the numbers of transient contacts that are above one are reported in Fig. 6b; see also the legend of this figure); O5 makes three transient contacts with His87 (seen in Fig. 6b as three CH
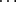
O contacts between His87 and the SO_2_ group). It is also noteworthy in Fig. 6a that Ethe and Tol participate in several transient contacts. In Fig. 6b, one can see that Tol makes 12 contacts with Tyr82 and His87 while, despite its small size, Ethe makes 10 contacts of various types with Gln53, Val55, Ile56, and Tyr82. The transient nature of these contacts results from the flexibility of these peripheral moieties. The flexibility is brought by the dihedral rotations and by the rotational oscillations that are associated with an energy change that is lower than 1 kcal/mol around each of the bonds N7-S8 and S8-C17 for Tol, and O3-C31 for Ethe, as can be seen from the CHARMM force field parameters derived for this ligand [16]. The amplitude of rotation around these bonds is ∼90°, ∼75°, and ∼200°, respectively [16]. In Ethe, the very mobile atom C32 interacts with the backbone atoms of the residues 53 through 56 and also with the side chains of Gln53 and Ile56 (1.7 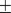 0.6, 0.9 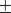 0.5, 2.1 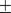 1.0, and 1.7 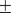 0.7 contacts between C32 and these four residues, respectively). Although less mobile than C32, the atom O2 of the Ethe moiety makes several transient contacts with Tyr82. As can be seen in Fig. 7, Tol is surrounded by three groups of atoms and forms numerous transient contacts with the side chains of Tyr82 and His87 as well as intramolecular contacts with *i*Bu (3.9 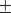 1.9, 7.9 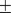 2.9, and 2.1 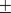 0.5 contacts, respectively). In Fig. 6a, one can see that Tol makes almost all its contacts with Tyr82 and His87 and only very few contacts with Thr85 and Gly86. The large mobility of the side chain of His87, as can be inferred from the corresponding large RMSFs in Fig. 5, is instrumental in favoring transient contacts. For the 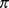
–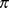 interactions between Tol and the side chains of Tyr82 and His87, we can hypothesize that the methyl group of the atom C23 (Fig. 1a) is responsible for an electronic enrichment on both sides of the Tol ring, resulting in an enhanced 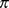
–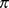 interactions with the rings of Tyr82 and His87. It should also be the case for any other donor group around this Tol ring in a ligand analogue.

In the intermediate IS308, we found that Tyr82 of FKBP12 makes the highest number of intermolecular contacts (12.9 vs. 11.3 for His87). As can be seen in Fig. 6b, the residue Tyr82 makes four contacts with each of the groups of atoms Tol, C2SN7/C1O1N1, and Ethe while His87 makes eight contacts with Tol and three with the SO_2_ group. Hence, the contacts of Tyr82 are largely spread over the various groups of atoms of the ligand (except for the remote *i*Bu that makes only 0.2 contact with Tyr82; Fig. 6a), and are of various types, involving either its cycle (through 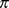
–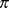 or CH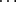 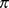 interactions) or its hydroxyl group (CH
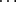
O or OH
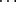
O; Fig. 6b). In particular, the side chain of Tyr82 makes 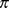
–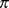 interactions with the Tol moiety. A similar result between Tyr82 and the Ph1 moiety of the ligand **8** was found in our previous study where we have also emphasized the importance of this interaction in the recognition process [13]. Our intermediates IS8 and IS308 exemplify the versatile role of Tyr82 in the recognition process of the FKBP12 binding partners: this protein residue can not only form various types of contact, but it can also form 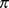
–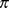 contacts with aromatic rings that are not equivalent in terms of relative positions in the two ligands. Indeed, Tol in **308** is the counterpart of *i*Pe in **8** (not Ph1) when one compares the two ligand positions in the bound states [7], [8] (Fig. 1). Our results are thus consistent with the general observations that the residue Tyr is the most effective for mediating molecular recognition [40].

Another interesting aspect of IS308 is the above-mentioned intramolecular interactions between the interchangeable atoms C25/C26 of *i*Bu and all the atoms of the Tol cycle. In IS308, but also in the free state, two of these intramolecular contacts are formed (2.08 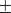 0.52 and 1.88 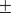 0.23 contacts, respectively) whereas few of them are monitored in CS308 (0.44 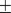 0.57). In CS308, the *i*Bu moiety prefers the interaction with the side chains of the residues Phe46, Glu54, Val55, and His87. The situation is also similar in IS8 since intramolecular contacts can be found between the methyl carbon C13 and the Ph2 moiety. Another similarity thus emerges between the two ligands **308** and **8**: in each case and both in the free state and in the intermediate, two peripheral moieties of the ligand interact with each other, namely, a methyl group makes many interchangeable contacts with the equivalent CH groups of an aromatic ring (C25/C26
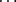
Tol or C13
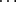
Ph2). Hence, in IS308 the ligand retains its intramolecular contacts as in its free state (dashed blue line in Fig. 6a) while the moieties Tol and *i*Bu are also interacting with the protein side chains (Figs. 6a and 6b), contributing to an enthalpic gain.

Finally, it should be mentioned that the central ring C2SN7 only barely interacts with the protein in IS308 and makes about only one third of the contacts it makes in the complexed state (5.3 contacts that are spread over eight residues in comparison with 15.6 contacts in CS308). In Fig. 6a, the contacts made by the ring C2SN7 with six protein residues can be seen on the map with the corresponding numbers of contacts that are below 1.5; the contacts with the two other residues Glu54 and Ile56 can not be seen on the map since the number is about 0.1. In contrast, in CS308 only four residues (Tyr26, Phe46, Trp59, and Tyr82) are responsible for 14 of the 15.6 contacts made by the ring C2SN7.

## Discussion

We characterized an intermediate state along the binding pathway of ligand **308** to FKBP12. The set of molecular dynamics simulations indicate consistent results for all the structural characteristics such as the structure of the protein, the position of the ligand or the type of intermolecular contacts. The stability of the ligand at the entrance of the binding pocket is due to the numerous transient contacts as well as to a few permanent contacts. The transient nature of the majority of the contacts formed could help to avoid a trapped intermediate state along the complexation pathway.

The mobility due to torsions or to rotational oscillations of the peripheral moieties, along with their peculiar geometry (symmetry of the aromatic ring of Tol, three-fold symmetry of the methyl carbon C32, and equivalence between the C25 and C26 methyl groups of *i*Bu), favors transient contacts between interchangeable atoms of the ligand and some protein residues. Recently, the importance of this type of transient contacts was shown in a study on the contributions in the configurational entropy of the side chains of residues during the formation of the complex PKA/AKAP [41]. The authors have found that the affinity between both proteins increases with the number of transient contacts formed between equivalent atoms of the hydrophobic side chains of both proteins. The authors showed that this number of equivalent transient contacts (also called alternative in their study) increases with the configurational entropy. Although their conclusions were drawn from the analysis of one native protein-protein complex, it is likely that, also in the case of IS8 and IS308, the significant number of transient contacts could help the ligand to retain at least a part of the configurational entropy it has in the free state while the nascent interactions would contribute to an enthalpic gain. This assertion is further supported by the fluctuations of the ligand calculated in the intermediates and that are similar to those in the free state (Fig. 5).

Another important feature of the intermediate states IS308 and IS8 is the distortion of the 80s loop. In the respective complexed state, the ligand **308** or **8** is deeply buried in the binding site of FKBP12: the cycle C2SN7 or C2N7 is buried in the main pocket while the Tol moiety of **308** or the methyl group of the atom **8**-C13 is buried in the small side-cavity formed by the four bulkiest side chains of the 80s loop (residues Tyr82, His87, Ile90, and Ile91). On the basis of these observations, a binding mechanism between the ligand **308** or **8** and a rigid protein FKBP12 that would occur without any binding intermediate would be prevented by a large steric hindrance for the entrance into the binding pocket. The results gathered from the docking of the ligand FK506 to a rigid protein FKBP12 [42] are consistent with the above analysis; it is worth considering the results of such a study since FK506 and the ligands **308**/**8** share an analogous binding mode with nearly superimposable binding regions. In this rigid docking study, the author failed to identify a geometry close to the experimentally reported structure of FKBP12-FK506 because of unfavorable steric overlaps. Interestingly, this author has found that the relaxation of the protein in the 80s loop region helps to accommodate the ligand in the binding pocket. Hence, during the binding process of FK506 or related ligands such as **308** or **8**, a displacement of the 80s loop would facilitate the binding to FKBP12. This hypothesis is further supported by a comparison between solution structures of unliganded FKBP12 and a crystal structure of FKBP12-FK506 [38], [39]. In this study, Ivery and Weiler have concluded that, during the binding, the loop should move as a unit and, in particular, the residues His87 and Ile90 should undergo a large positional shift. The above hypothesis is also supported by results gathered on the unliganded form of FKBP12: the 80s loop has been shown to be very mobile, especially in the 82–90 region [43]. NMR data also indicate that this loop is sampling several configurations [44]. Taken together, the above observations thus strongly suggest that, during the binding, the 80s loop is actually undergoing conformational transitions to facilitate the entrance of the ligand by reducing the steric hindrance, as observed in our binding intermediate model. The reduction of the steric hindrance corresponds to a decreased entropic penalty for the overall energy barrier of complexation because of the fewer structural constraints for the ligand in this case than in a hypothetical one-step mechanism in which both the core and the Tol or *i*Pe moiety of the ligand have to fit simultaneously into the main pocket and the small side-cavity, respectively.

The formation and the stability of the binding intermediates IS308 and IS8 result from the three following contributions: (i) an entropic penalty due to the steric hindrance with the 80s loop that is reduced owing to its displacement, (ii) an enthalpic gain due to the numerous transient and few permanent interactions in the intermediate, and (iii) the above-discussed configurational entropy that increases with the number of transient contacts and that is hypothesized to be large since most of the numerous contacts made in IS are transient. Since these three energetic considerations contribute to increasing the stability of the intermediate, as a corollary, the dissociation rate constant 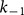 of this intermediate should be decreased, resulting in an increase of the affinity constant 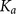 (equations 5 and 2). Hence, the analysis of our binding intermediate throws light on the binding properties of the high-affinity ligand **308**.

A relationship between the characteristics of the binding intermediate and their incidence on the association rate constant 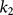 of the intermediate is more difficult to predict. However, if the intermediate state IS is close in energy and structure to the transition state TS found between IS and the complexed state CS, then a direct incidence can be expected. Indeed, the latter condition ensures that the following reasoning made on TS is transferable to IS. Under this condition, two major forces could drive TS, and thus IS also, to CS. The first force has an entropic nature and is the desolvation, between TS and CS, of the hydrophobic cycle C2N7 in **8** or C2SN7 in **308** that is still solvated in IS/TS and that only barely interacts with the protein (Fig. 6 and ref. [13]); in CS, however, this cycle is deeply buried in the main pocket. The second force has an enthalpic nature and corresponds to the formation of the two hydrogen bonds between TS and CS and that are only nascent in IS/TS (hydrogen bonds formed by O1 and O2 in **308** and by O2 and O3 in **8**). Hence, if the above condition is satisfied, our model of IS would come closest to explaining both the two recurrent oxygen atoms and the prevalence of hydrophobic counterparts in various FKBP12 ligands as substituents for C2SN7, as these structural features are linked to hydrogen bond formation and desolvation that can drive the intermediate to the final complex. Under the above-defined condition, these forces would therefore increase 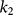 and, thus, the constant 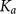. Our model may therefore also explain why various substituents are found for C2SN7 (five- or six-membered ring or open alkyl forms), since hydrophobicity seems to be the only common features of this part of the ligand.

**Figure 8:**
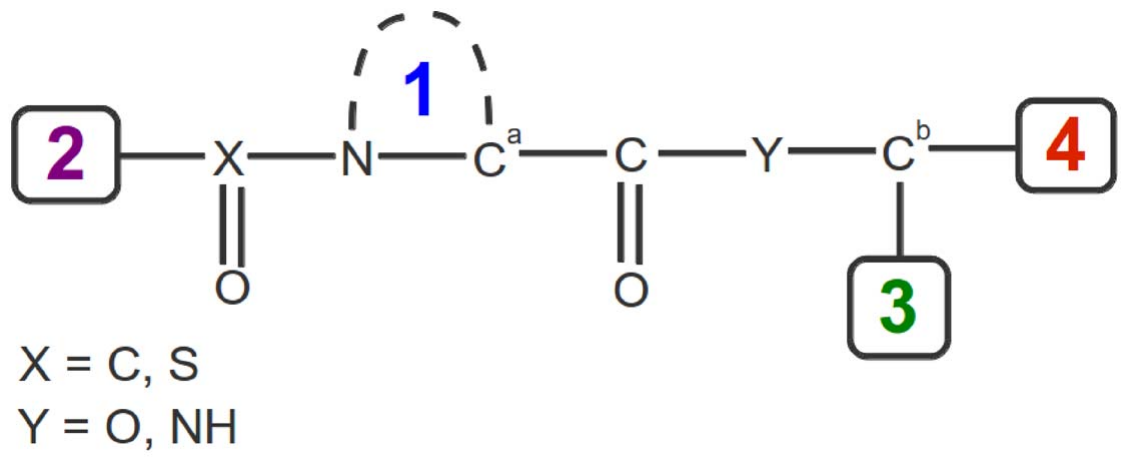
Structure of a typical high-affinity ligand of FKBP12 with the peripheral groups of atoms labelled 2 to 4. The central group of atoms 1 is often a five- or six-membered ring; however, open alkyl forms for this central group can also be found [6].

From the analysis of our intermediate model, we are able to provide clues regarding the nature of the substituents 1 to 4, as depicted in Fig. 8, that are determining for high binding properties. The group labelled 1 should be a hydrophobic group for the desolvation between IS and CS (under the above-defined condition only). A ring is not a prerequisite since binding to an isoform of FKBP occurs with an alkyl chain linked only to C*^α^* (presumably to FKBP52 whose N-terminal FK506 binding domain is very similar in sequence and structure to FKBP12 [6], [45]). However, the benefit of having a five- or six-membered ring for the group 1 is to constrain in space the position of the two vicinal carbonyl whose oxygen atoms act as anchors in the binding process. The group 2 in Fig. 8 should be mobile to ensure transient contacts with His87 and Tyr82, and an axial symmetry for this group, as in phenyl or *t*-butyl groups (or pseudo-symmetry as for *i*Pe in ligand **8**), allows the transient contacts to be interchangeable (note that the 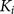 constants that have been measured for a series of ligands with *t*-butyl as group 2 are systematically lower than those measured with the *i*Pe group as substituent [46]). In their work, Zhao *et al.* have also found that *t*-butyl as group 2 exhibited better binding ability to the FKBP isoform than a methyl-butyl group [6]. The group 3 should be bulky (to allow intramolecular contacts with the group 2) and symmetric (for interchangeable contacts with the group 2 or with the protein residues), as in the ligands **8** and **308** where all the contacts formed are equivalent owing to the rotation of the phenyl or *i*Bu groups, respectively [13]. The structure of the group 4 in Fig. 8 interacts with Tyr82 in the intermediate, either with the Tyr cycle (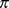
–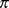 or CH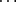 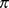) or with its hydroxyl group (OH
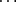
O). Indeed, most of the known high-affinity ligands of FKBP12 have an aromatic ring (phenyl, pyridyl or trimethoxyphenyl ring) linked by an ethyl ‘arm’ to the atom C*^b^* (Fig. 8).

Hence, the analysis of our intermediates provide a rationale for explaining the diversity of the core and non-core structures found in various high-affinity ligands of FKBP12.

## Conclusion

We characterized a binding intermediate state IS308 between the protein FKBP12 and a high-affinity ligand **308**. An interesting aspect of IS308 concerns the 80 s loop of the protein that is distorted, mainly in its tip region. This distortion leads to the exposure of the side-cavity formed by the four bulkiest side chains of this loop to the incoming ligand, thereby facilitating the accessibility to the bound state. The ligand has a stable and consistent position throughout all of the simulations and is found at a distance of the protein that allows only seven permanent interactions to be formed (among 71 contacts), mainly by the atoms of the ligand core. Besides, our analysis of the binding intermediate in terms of contacts throws some light on the role played by the peripheral moieties in this binding step: Ethe and Tol are major transient contacts makers. The transient nature of these interactions seems crucial for the affinity of binding since it has been shown, in the context of the protein-protein interactions, that their quantity determines the affinity [41]. The dynamic aspect of the interactions formed by the peripheral moieties also helps to avoid a trapped state along the binding pathway and offers the possibility of fine-tuning the specificity of recognition.

As the peripheral groups are mainly involved in transient interactions, we found that O1 and O5 are of particular importance for the stability of the ligand in IS308 since they act as two anchors, forming permanent contacts with Ile56 and Ile90. Interestingly, the same result was found in the case of the dissimilar ligand **8** [13]. Thus, by stabilizing the intermediate state IS308 (or IS8), the transient and anchoring interactions contribute to decreasing its dissociation rate 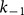, and thus to increasing the affinity constant 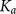. For the core and non-core moieties of high-affinity ligands of FKBP12, these results can thus explain the prevalence of (i) the two oxygen atoms found in carbonyl or sulfonyl groups of dissimilar core structures, and of (ii) symmetric or pseudo-symmetric mobile groups of atoms found as non-core moieties.

## Supporting Information

S1 Text **Definition of the sets C1, C2 and C5 of NOE restraints.**
(PDF)

S1 Table **Summary of the NOE restraints that were used to perform the MD simulations.** The use of these restraints resulted with a good agreement with the experimental structure (in terms of protein and 80 s loop structure, interatomic contacts, and RMSDs). See the definition of the sets of restraints C3 and C4 in S2 and S3 Tables, respectively.
(PDF)

S2 Table
**Set of NOE restraints C3.** The values of R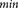 and R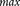 that were used in the MD simulations with NOE restraints correspond to 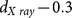 Å et 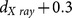 Å, respectively, where 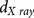 is the experimental distance measured in the crystal structure in Å.(PDF)

S3 Table
**Set of NOE restraints C4.** The values of R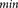 and R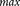 that were used in the MD simulations with NOE restraints correspond to 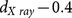 Å et 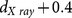 Å where 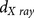 is the experimental distance measured in the crystal structure in Å.(PDF)

S4 Table
**Average distances between the respective center of mass of the four ligand moieties and that of the binding pocket.** The average 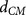 values (in Å) calculated from the subset of LD simulations that passed the acceptance criteria (see text) are given along with the ensemble averages. Exp. corresponds to the X-ray structure. For comparison, the ensemble average values for CS308, calculated from the SBD simulations of the complexed state, are also reported.(PDF)

S5 Table **RMSDs of the ligand and of its four moieties from the respective X-ray structural positions.** The average values (in Å) calculated from the subset of LD simulations that passed the acceptance criteria (see text) are given along with the ensemble averages (only the heavy atoms were considered). The alignment was based upon all the protein C*_α_* atoms. For comparison, the ensemble average values for CS308, calculated from the SBD simulations of the complexed state, are also reported.
(PDF)

S6 Table **Time average and ensemble average distances (Å) for the most persistent contacts between the protein and the ligand.** A persistent contact was considered whenever the corresponding contact frequency was higher than 50% in at least one simulation. The reported fluctuation range (Å) represents the minimum and the maximum of the fluctuations in the simulation set.
(PDF)

## References

[1] DeCenzo MT, Park ST, Jarrett BP, Aldape RA, Futer O, et al (1996) FK506-binding protein mutational analysis: defining the active-site residue contributions to catalysis and the stability of ligand complexes. Prot Eng 9:173–180. 10.1093/protein/9.2.173 9005438

[2] Bierer BE, Mattila PS, Standaert RF, Herzenberg LA, Burakoff SJ, et al (1990) Two distinct signal transmission pathways in T lymphocytes are inhibited by complexes formed between an immunophilin and either FK506 or rapamycin. Proc Natl Acad Sci U S A 87:9231–9235. 2123553 10.1073/pnas.87.23.9231 PMC55138

[3] Liu J, Farmer JD, Friedman J, Weissman I, Schreiber SL (1991) Calcineurin is a common target of cyclophilin-cyclosporin A and FKBP-FK506 complexes. Cell 66:807–815. 1715244 10.1016/0092-8674(91)90124-h

[4] Jiang BH, Liu LZ (2008) Role of mTOR in anticancer drug resistance: Perspectives for improved drug treatment. Drug Resist Updates 11:63–76. 10.1016/j.drup.2008.03.001 PMC2519122 18440854

[5] Herdegen T, Fischer G, Gold BG (2000) Immunophilin ligands as a novel treatment for neurological disorders. Trends Pharmacol Sci 21:3–5. 10712163 10.1016/s0165-6147(99)01407-8

[6] Zhao L, Liu H, Wang L, Li S (2006) Modeling and synthesis of non-cyclic derivatives of GPI-1046 as potential FKBP ligands with neurotrophic properties. Bioorg Med Chem Lett 16:4385–4390. 16753298 10.1016/j.bmcl.2006.05.044

[7] Holt D, Luengo J, Yamashita D, Oh H, Konialian A, et al (1993) Design, synthesis, and kinetic evaluation of high-affinity FKBP ligands and the X-ray crystal structures of their complexes with FKBP12. J Am Chem Soc 115:9925–9938.

[8] Sun F, Li P, Ding Y, Wang L, Bartlam M, et al (2003) Design and structure-based study of new potential FKBP12 inhibitors. Biophys J 85:3194–3201. 14581219 10.1016/S0006-3495(03)74737-7 PMC1303595

[9] Yang W, Rozamus LW, Narula S, Rollins CT, Yuan R, et al (2000) Investigating protein-ligand interactions with a mutant FKBP possessing a designed specificity pocket. J Med Chem 43:1135–1142. 10737745 10.1021/jm9904396

[10] Lamb ML, Jorgensen, W L (1998) Investigations of neurotrophic inhibitors of FK506 binding protein via monte carlo simulations. J Med Chem 41:3928–3939. 9767630 10.1021/jm980062o

[11] Wang J, Deng Y, Roux B (2006) Absolute binding free energy calculations using molecular dynamics simulations with restraining potentials. Biophys J 91:2798–2814. 16844742 10.1529/biophysj.106.084301 PMC1578458

[12] Xu Y, Wang R (2006) A computational analysis of the binding affinities of FKBP12 inhibitors using the MM-PB/SA method. Proteins Struct Funct Genet 64:1058–1068. 16838311 10.1002/prot.21044

[13] Olivieri L, Gardebien F (2011) Molecular dynamics simulations of a binding intermediate between FKBP12 and a high-affinity ligand. J Chem Theory Comput 7:725–741. 26596305 10.1021/ct100394d

[14] Mittag T, Schaffhausen B, Günther UL (2004) Tracing kinetic intermediates during ligand binding. J Am Chem Soc 126:9017–9023. 15264834 10.1021/ja0392519

[15] MacKerell Jr AD, Bashford D, Bellott M, Dunbrack Jr RL, Evanseck JD, et al (1998) All-atom empirical potential for molecular modeling and dynamics studies of proteins. J Phys Chem B 102:3586–3616. 24889800 10.1021/jp973084f

[16] Olivieri L, Gardebien F (2014) Classical force field parameters for two high-affinity ligands of FKBP12. J Mol Graph Model 49:118–128. 24657432 10.1016/j.jmgm.2014.02.003

[17] Babine RE, Bender SL (1997) Molecular recognition of protein-ligand complexes: Applications to drug design. Chem Rev 97:1359–1472. 11851455 10.1021/cr960370z

[18] Frisch MJ, Trucks GW, Schlegel HB, Scuseria GE, Robb MA, et al. (2004) Gaussian03. Gaussian, Inc., Wallingford, CT.

[19] Schmidt MW, Baldridge KK, Boatz JA, Elbert ST, Gordon MS, et al (1993) General atomic and molecular electronic structure system. J Comput Chem 14:1347–1363.

[20] Pettersen EF, Goddard TD, Huang CC, Couch GS, Greenblatt DM, et al (2004) UCSF Chimera–a visualization system for exploratory research and analysis. J Comput Chem 25:1605–1612. 15264254 10.1002/jcc.20084

[21] Brooks BR, Brooks III CL, Mackerell AD Jr, Nilsson L, Petrella RJ, et al (2009) CHARMM: The biomolecular simulation program. J Comput Chem 30:1545–1614. 19444816 10.1002/jcc.21287 PMC2810661

[22] Ryckaert JP, Ciccotti G, Berendsen HJC (1977) Numerical integration of the cartesian equations of motion of a system with constraints: molecular dynamics of n-alkanes. J Comput Phys 23:327–341.

[23] Lemons DS, Gythiel A (1997) Paul langevin's 1908 paper ‘On the theory of brownian motion’. Am J Phys 65:1079–1081.

[24] Lazaridis T, Karplus M (1999) Effective energy function for proteins in solution. Proteins Struct Funct Genet 35:133–152. 10223287 10.1002/(sici)1097-0134(19990501)35:2<133::aid-prot1>3.0.co;2-n

[25] Szep S, Park S, Boder ET, Van Duyne GD, Saven JG (2009) Structural coupling between FKBP12 and buried water. Proteins Struct Funct Bioinf 74:603–611. 10.1002/prot.22176 PMC2629809 18704951

[26] van Duyne GD, Standaert RF, Karplus PA, Schreiber SL, Clardy J (1993) Atomic structures of the human immunophilin FKBP12 complexes with FK506 and rapamycin. J Mol Biol 229:105–124. 7678431 10.1006/jmbi.1993.1012

[27] Brünger A, Brooks CL III, Karplus M (1984) Stochastic boundary conditions for molecular dynamics simulations of ST2 water. Chem Phys Lett 105:495–500.

[28] Paci E, Caflisch A, Plückthun A, Karplus M (2001) Forces and energetics of hapten-antibody dissociation: a biased molecular dynamics simulation study. J Mol Biol 314:589–605. 11846569 10.1006/jmbi.2001.5103

[29] Swegat W, Schlitter J, Krüger P, Wollmer A (2003) MD simulation of protein-ligand interaction: formation and dissociation of an insulin-phenol complex. Biophys J 84:1493–1506. 12609856 10.1016/S0006-3495(03)74962-5 PMC1302723

[30] Huang H, Ozkirimli E, Post C (2009) Comparison of three perturbation molecular dynamics methods for modeling conformational transitions. J Chem Theory Comput 5:1304–1314. 10.1021/ct9000153 PMC2731424 20161143

[31] Curcio R, Caflisch A, Paci E (2005) Change of the unbinding mechanism upon a mutation: A molecular dynamics study of an antibody-hapten complex. Protein Sci 14:2499–2514. 16195542 10.1110/ps.041280705 PMC2253310

[32] Li A, Daggett V (1998) Molecular dynamics simulation of the unfolding of barnase: characterization of the major intermediate. J Mol Biol 275:677–694. 9466940 10.1006/jmbi.1997.1484

[33] Peters GH, Bywater RP (1999) Computational analysis of chain flexibility and fluctuations in *Rhizomucor miehei* lipase. Prot Eng 12:747–754. 10.1093/protein/12.9.747 10506284

[34] Teplyakov A, Sebastiao P, Obmolova G, Perrakis A, Brush GS, et al (1996) Crystal structure of bacteriophage T4 deoxynucleotide kinase with its substrates dGMP and ATP. EMBO J 15:3487–3497. 8670851 PMC451945

[35] Hornak V, Okur A, Rizzo RC, Simmerling C (2006) HIV-1 protease flaps spontaneously open and reclose in molecular dynamics simulations. Proc Natl Acad Sci U S A 103:915–920. 16418268 10.1073/pnas.0508452103 PMC1347991

[36] Burkhard P, Taylor P, Walkinshaw MD (2000) X-ray structures of small ligand-FKBP complexe provide an estimate for hydrophobic interaction energies. J Mol Biol 295:953–962. 10656803 10.1006/jmbi.1999.3411

[37] Rosen MK, Yang D, Martin PK, Schreiber SL (1993) Activation of an inactive immunophilin by mutagenesis. J Am Chem Soc 115:821–822.

[38] Ivery MTG, Weiler L (1997) Modeling the interaction between FK506 and FKBP12: a mechanism for formation of the calcineurin inhibitory complex. Bioorg Med Chem Lett 5:211–232. 10.1016/s0968-0896(96)00229-5 9061187

[39] Wilson KP, Yamashita MM, Sintchak MD, Rotstein SH, Murcko MA, et al (1995) Comparative X-ray structures of the major binding protein for the immunosuppressant FK506 (tacrolimus) in unliganded form and in complex with FK506 and rapamycin. Acta Cryst D51:511–521. 10.1107/S0907444994014514 15299838

[40] Koide S, Sidhu SS (2009) The importance of being Tyrosine: lessons in molecular recognition from minimalist synthetic binding proteins. ACS Chem Biol 4:325–334. 19298050 10.1021/cb800314v PMC2829252

[41] Chang CA, McLaughlin WA, Baron R, Wang W, McCammon JA (2008) Entropic contributions and the influence of the hydrophobic environment in promiscuous protein-protein association. Proc Natl Acad Sci U S A 105:7456–7461. 18495919 10.1073/pnas.0800452105 PMC2391134

[42] Zacharias M (2004) Rapid protein ligand docking using soft modes from molecular dynamics simulations to account for protein. Proteins Struct Funct Genet 54:759–767. 14997571 10.1002/prot.10637

[43] Michnick SW, Rosen MK, Wandless TJ, Karplus M, Schreiber SL (1991) Solution structure of FKBP, a rotamase enzyme and receptor for FK506 and rapamycin. Science 252:836–839. 1709301 10.1126/science.1709301

[44] Rosen MK, Schreiber SL (1992) Natural products as probes of cellular function: studies of immunophilins. Angew Chem Int Ed 31:384–400.

[45] Davies TH, Sánchez ER (2005) FKBP52. Int J Biochem Cell Biol 37:42–47. 15381148 10.1016/j.biocel.2004.03.013

[46] Hamilton GS, Huang W, Connolly MA, Ross DT, Guo H, et al (1997) FKBP12-binding domain analogues of FK506 are potent, nonimmunosuppressive neurotrophic agents in vitro and promote recovery in a mouse model of Parkinson's disease. Bioorg Med Chem Lett 7:1785–1790.

