# Supplementary material for: Structure-Affinity Properties of a High-Affinity Ligand of FKBP12 Studied by Molecular Simulations of a Binding Intermediate
Source: PLoS One. 2014 Dec 12;9(12):e114610. doi: 10.1371/journal.pone.0114610 (PMC4264844; doi:10.1371/journal.pone.0114610)
Supplement: S1 Text — Definition of the sets C1, C2 and C5 of NOE restraints. (PDF) [file pone.0114610.s001.pdf]

Text S1. Definition of the sets C1, C2 and C5 of NOE restraints

To avoid the distortion of the 80s loop during the minimizations and the subsequent LD simulations, the distances between Thr96-O$\cdots$Glu31-H, Glu31-$O^{\epsilon 2}\cdots$Thr96-$O^{\gamma 1}$, Glu31-$O^{\epsilon 1}\cdots$Thr96-$O^{\gamma 1}$, Glu31-$O^{\epsilon 2}\cdots$Thr96-N, and Ile90-O$\cdots$Lys34-N^{ζ} (set C1 of NOE restraints) were forced to remain above their values measured in the crystal (3.3, 8.7, 8.0, 7.9, and 10.64 Å, respectively) by using CHARMM NOE restraints. For all the present and following NOE restraints, the KMIN, KMAX, and FMAX were chosen at 100.0, 100.0, and 500.0 kcal/mol/$Å^2$, respectively. In addition, a NOE restraint was applied during the minimization to keep the distance between the two backbone atoms Tyr82-O and Thr85-H between 2.8 and 3.5 Å (set C2 of NOE restraints).

The set C5 corresponds to all the pairs of atoms between the protein and the ligand that are separated by less than 5 Å in the crystallographic structure with PDB code 1J4I.
