# Supplementary material for: Structure-Affinity Properties of a High-Affinity Ligand of FKBP12 Studied by Molecular Simulations of a Binding Intermediate
Source: PLoS One. 2014 Dec 12;9(12):e114610. doi: 10.1371/journal.pone.0114610 (PMC4264844; doi:10.1371/journal.pone.0114610)
Supplement: S1 Table — Summary of the NOE restraints that were used to perform the MD simulations. The use of these restraints resulted with a good agreement with the experimental structure (in terms of protein and 80 s loop structure, interatomic contacts, and RMSDs). See the definition of the sets of restraints C3 and C4 in S2 and S3 Tables, respectively. (PDF) [file pone.0114610.s002.pdf]

Table S1. Summary of the NOE restraints that were used to perform the MD simulations. The use of these restraints resulted with a good agreement with the experimental structure (in terms of protein and 80s loop structure, interatomic contacts, and RMSDs). See the definition of the sets of restraints C3 and C4 in tables S2 and S3, respectively.

MD Step	LD	SBD
Heating	C1, C2, C3, C4	C1, C2, C4, C5
Equilibration 1	C1, C2, C3, C4	C1, C2 C4, C5
Equilibration 2	C1, C2, C3	$\emptyset$
Production	C1, C2, C3	$\emptyset$
