# Supplementary material for: Structure-Affinity Properties of a High-Affinity Ligand of FKBP12 Studied by Molecular Simulations of a Binding Intermediate
Source: PLoS One. 2014 Dec 12;9(12):e114610. doi: 10.1371/journal.pone.0114610 (PMC4264844; doi:10.1371/journal.pone.0114610)
Supplement: S2 Table — Set of NOE restraints C3. The values of R and R that were used in the MD simulations with NOE restraints correspond to Å et Å, respectively, where is the experimental distance measured in the crystal structure in Å. (PDF) [file pone.0114610.s003.pdf]

Table S2. Set of NOE restraints C3. The values of R_{min} and R_{max} that were used in the MD simulations with NOE restraints correspond to $d_{Xray} - 0.3$ Å et $d_{Xray} + 0.3$ Å, respectively, where d_{Xray} is the experimental distance measured in the crystal structure in Å.

Atom pair	d_{Xray}
49-O···54-N $^{(a)}$	5.2
49-O···Glu60-O$^{\epsilon 1}$ $^{(a)}$	4.1
54-N···Glu60-O$^{\epsilon 1}$ $^{(a)}$	5.1
57-N···81-O $^{(b)}$	5.7
69-O···102-O$^{\epsilon 2}$ $^{(b)}$	4.7
82-N···95-O $^{(b)}$	5.3
83-O···92-O $^{(b)}$	4.8

[a]Constraint used to mimic the presence of a water molecule, as determined by Szep *et al.* [**?**]

[b]Constraint used to mimic the presence of a water molecule, as determined by Van Duyne *et al.* [**?**]
