# Supplementary material for: Structure-Affinity Properties of a High-Affinity Ligand of FKBP12 Studied by Molecular Simulations of a Binding Intermediate
Source: PLoS One. 2014 Dec 12;9(12):e114610. doi: 10.1371/journal.pone.0114610 (PMC4264844; doi:10.1371/journal.pone.0114610)
Supplement: S3 Table — Set of NOE restraints C4. The values of R and R that were used in the MD simulations with NOE restraints correspond to Å et Å where is the experimental distance measured in the crystal structure in Å. (PDF) [file pone.0114610.s004.pdf]

Table S3. Set of NOE restraints C4. The values of R_{min} and R_{max} that were used in the MD simulations with NOE restraints correspond to $d_{Xray} - 0.4$ Å et $d_{Xray} + 0.4$ Å where d_{Xray} is the experimental distance measured in the crystal structure in Å.

Atom pair	d_{Xray}
Glu5-$O^{\epsilon 2}$···Thr75-$H^{\gamma 1}$ $^{(a)}$	7.5
Asp11-$O^{\delta 1}$···Arg13-H $^{(a)}$	2.1
Asp11-$O^{\delta 1}$···Thr14-H $^{(a)}$	1.8
Tyr26-O^{η}···Arg42-$H^{\eta 22}$ $^{(a)}$	2.0
Thr27-$O^{\gamma 1}$···Ser38-H^{γ} $^{(a)}$	3.0
Asp32-$O^{\delta 1}$···Lys34-H $^{(a)}$	2.1
Asp37-$O^{\delta 1}$···Arg42-$N^{\eta 1}$ $^{(a)}$	3.2
Asp37-$O^{\delta 2}$···Tyr26-H^{η} $^{(a)}$	1.8
Asp37-$O^{\delta 2}$···Arg42-$N^{\eta 1}$ $^{(a)}$	3.1
Asp41-$O^{\delta 2}$···Lys35-N^{ζ} $^{(a)}$	2.8
Glu54-$O^{\epsilon 1}$···Lys52-N^{ζ} $^{(a)}$	4.1
Glu61-$O^{\epsilon 2}$···Arg57-H^{ϵ} $^{(a)}$	2.3
Glu61-$O^{\epsilon 2}$···Arg57-$H^{\eta 21}$ $^{(a)}$	2.8
Glu61-$O^{\epsilon 1}$···Tyr80-H^{η} $^{(a)}$	4.3

[a]Constraint used to reduce the fluctuations of the residues involved.

[b]Constraint used to avoid the distortion of the 80s loop.

Atom pair	d_{Xray}
Ser77-O···Tyr80-H $^{(b)}$	2.2
Pro78-O···Gly83-H $^{(b)}$	1.8
Asp79-$O^{\delta 1}$···Ser77-H^{γ} $^{(a)}$	1.8
Tyr82-O···Gly86-H $^{(b)}$	1.9
Gly83-O···His94-H $^{(b)}$	1.7
His87-O···Ile90-H $^{(b)}$	2.3
His87-O···Ile91-H $^{(b)}$	1.8
Ile91-O···His87-H $^{(b)}$	1.7
**308**-O1···Ile56-H $^{(c)}$	1.9
**308**-H1···Tyr82-O^{η} $^{(c)}$	2.6
**308**-O2···Tyr82-O^{η}–H $^{(c)}$	1.7

[c]These constaints were instrumental in achieving agreement with the intermolecular distances measured in the crystal.
