# Supplementary material for: Structure-Affinity Properties of a High-Affinity Ligand of FKBP12 Studied by Molecular Simulations of a Binding Intermediate
Source: PLoS One. 2014 Dec 12;9(12):e114610. doi: 10.1371/journal.pone.0114610 (PMC4264844; doi:10.1371/journal.pone.0114610)
Supplement: S4 Table — Average distances between the respective center of mass of the four ligand moieties and that of the binding pocket. The average values (in Å) calculated from the subset of LD simulations that passed the acceptance criteria (see text) are given along with the ensemble averages. Exp. corresponds to the X-ray structure. For comparison, the ensemble average values for CS308, calculated from the SBD simulations of the complexed state, are also reported. (PDF) [file pone.0114610.s005.pdf]

Table S4. Average distances between the respective center of mass of the four ligand moieties and that of the binding pocket. The average d_{CM} values (in Å) calculated from the subset of LD simulations that passed the acceptance criteria (see text) are given along with the ensemble averages. Exp. corresponds to the X-ray structure. For comparison, the ensemble average values for CS308, calculated from the SBD simulations of the complexed state, are also reported.

	d_{CM}				
	ligand	Ethe	*i*Bu	core	Tol
Exp.	3.24	6.07	5.16	1.34	4.50
CS308	3.77 ± 0.07	6.52 ± 0.11	6.11 ± 0.22	1.59 ± 0.09	5.04 ± 0.10
LD1	7.50 ± 0.26	7.95 ± 0.32	9.36 ± 0.32	6.04 ± 0.37	8.73 ± 0.36
LD2	7.45 ± 0.24	8.47 ± 0.37	9.48 ± 0.28	5.73 ± 0.27	8.41 ± 0.45
LD3	7.49 ± 0.34	8.47 ± 0.45	9.38 ± 0.41	5.77 ± 0.31	8.50 ± 0.37
LD4	7.87 ± 0.30	7.99 ± 0.37	9.31 ± 0.34	5.86 ± 0.43	10.19 ± 0.40
LD5	7.80 ± 0.40	7.53 ± 0.45	8.91 ± 0.78	7.26 ± 0.51	9.29 ± 0.66
LD6	7.19 ± 0.48	7.75 ± 0.65	8.77 ± 0.81	5.84 ± 0.43	8.61 ± 0.52
LD7	7.64 ± 0.54	8.25 ± 0.46	9.30 ± 0.44	5.83 ± 0.47	9.22 ± 0.97
LD8	7.58 ± 0.30	8.32 ± 0.60	9.05 ± 0.38	5.53 ± 0.26	9.55 ± 0.35
LD9	7.34 ± 0.39	7.75 ± 0.39	8.99 ± 0.34	5.22 ± 0.45	9.56 ± 0.55
LD10	8.14 ± 0.32	8.11 ± 0.48	9.65 ± 0.33	6.22 ± 0.42	10.49 ± 0.45
LD11	7.87 ± 0.28	8.99 ± 0.50	9.74 ± 0.32	6.12 ± 0.32	9.26 ± 0.38
⟨LD⟩	7.63 ± 0.27	8.14 ± 0.41	9.27 ± 0.30	5.95 ± 0.52	9.25 ± 0.68
