# Supplementary material for: Structure-Affinity Properties of a High-Affinity Ligand of FKBP12 Studied by Molecular Simulations of a Binding Intermediate
Source: PLoS One. 2014 Dec 12;9(12):e114610. doi: 10.1371/journal.pone.0114610 (PMC4264844; doi:10.1371/journal.pone.0114610)
Supplement: S5 Table — RMSDs of the ligand and of its four moieties from the respective X-ray structural positions. The average values (in Å) calculated from the subset of LD simulations that passed the acceptance criteria (see text) are given along with the ensemble averages (only the heavy atoms were considered). The alignment was based upon all the protein Cα atoms. For comparison, the ensemble average values for CS308, calculated from the SBD simulations of the complexed state, are also reported. (PDF) [file pone.0114610.s006.pdf]

Table S5. RMSDs of the ligand and of its four moieties from the respective X-ray structural positions. The average values (in Å) calculated from the subset of LD simulations that passed the acceptance criteria (see text) are given along with the ensemble averages (only the heavy atoms were considered). The alignment was based upon all the protein C_α atoms. For comparison, the ensemble average values for CS308, calculated from the SBD simulations of the complexed state, are also reported.

	RMSD				
	ligand	Ethe	*i*Bu	core	Tol
CS308	1.59 ± 0.12	1.77 ± 0.30	2.24 ± 0.39	1.01 ± 0.04	1.53 ± 0.46
LD1	6.84 ± 0.32	3.48 ± 0.24	7.36 ± 0.41	6.92 ± 0.40	8.32 ± 0.42
LD2	6.47 ± 0.27	3.71 ± 0.29	6.95 ± 0.39	6.43 ± 0.30	7.86 ± 0.49
LD3	6.28 ± 0.36	3.32 ± 0.52	6.37 ± 0.65	6.36 ± 0.33	7.75 ± 0.50
LD4	6.32 ± 0.33	2.82 ± 0.33	6.33 ± 0.42	6.37 ± 0.44	8.05 ± 0.44
LD5	7.08 ± 0.42	2.65 ± 0.41	6.21 ± 0.86	7.89 ± 0.61	8.45 ± 0.74
LD6	6.15 ± 0.40	2.67 ± 0.60	5.67 ± 0.85	6.54 ± 0.43	7.60 ± 0.61
LD7	6.87 ± 0.80	3.61 ± 0.82	6.98 ± 1.07	6.75 ± 0.55	8.78 ± 1.22
LD8	6.34 ± 0.30	3.48 ± 0.56	6.28 ± 0.46	6.10 ± 0.32	8.28 ± 0.42
LD9	5.71 ± 0.51	2.85 ± 0.39	6.01 ± 0.56	5.65 ± 0.54	7.17 ± 0.71
LD10	6.47 ± 0.39	3.15 ± 0.39	6.60 ± 0.39	6.57 ± 0.45	8.02 ± 0.72
LD11	6.39 ± 0.27	3.76 ± 0.37	6.47 ± 0.37	6.36 ± 0.32	7.90 ± 0.44
⟨LD⟩	6.45 ± 0.38	3.23 ± 0.42	6.47 ± 0.48	6.54 ± 0.56	8.02 ± 0.44
