# Supplementary material for: Structure-Affinity Properties of a High-Affinity Ligand of FKBP12 Studied by Molecular Simulations of a Binding Intermediate
Source: PLoS One. 2014 Dec 12;9(12):e114610. doi: 10.1371/journal.pone.0114610 (PMC4264844; doi:10.1371/journal.pone.0114610)
Supplement: S6 Table — Time average and ensemble average distances (Å) for the most persistent contacts between the protein and the ligand. A persistent contact was considered whenever the corresponding contact frequency was higher than 50% in at least one simulation. The reported fluctuation range (Å) represents the minimum and the maximum of the fluctuations in the simulation set. (PDF) [file pone.0114610.s007.pdf]

Table S6. Time average and ensemble average distances (Å) for the most persistent contacts between the protein and the ligand. A persistent contact was considered whenever the corresponding contact frequency was higher than 50% in at least one simulation. The reported fluctuation range (Å) represents the minimum and the maximum of the fluctuations in the simulation set.

Atom pair																					
FKBP12		Ligand	MD1	MD2	MD3	MD4	MD5	MD6	MD7	MD8	MD9	MD10	MD11	MD12	MD13	MD14	MD15	MD16	MD17	fluctuation range	MD	Std dev.
Phe46	C^{ζ}	S4	5.23	4.76	3.94	3.90	4.06	4.24	4.09	4.02	4.01	4.10	3.93	4.98	4.05	4.08	4.03	4.20	4.07	0.30-0.84	4.22	0.39
Gln53	O	C16	3.61	4.83	6.11	6.06	5.51	5.78	5.45	5.73	6.48	6.41	5.99	5.08	5.01	5.07	5.83	5.18	5.58	0.40-1.08	5.51	0.69
Glu54	O	O3	5.69	6.08	3.98	3.77	4.14	4.22	4.01	4.07	3.69	4.36	3.77	4.77	4.56	4.42	3.93	4.68	3.97	0.51-0.94	4.36	0.66
		C26	5.87	5.24	4.34	6.04	5.88	5.79	5.94	6.05	5.98	6.45	5.89	5.93	5.68	5.80	6.10	5.74	5.89	0.46-1.11	5.80	0.45
		C16	3.71	3.63	4.14	3.94	3.71	3.67	3.82	3.70	4.10	4.27	3.96	3.73	3.60	3.70	3.99	3.62	3.79	0.28-0.65	3.83	0.20
		C15	3.83	4.08	3.67	3.60	3.55	3.53	3.56	3.60	3.60	3.67	3.61	3.55	3.65	3.63	3.60	3.65	3.52	0.23-0.48	3.64	0.13
		N1	3.80	3.95	4.45	4.50	4.35	4.30	4.38	4.47	4.49	4.45	4.51	3.86	4.32	4.35	4.42	4.25	4.34	0.25-0.50	4.30	0.22
Val55	C	O3	5.49	5.50	4.09	3.93	4.03	4.05	3.95	4.09	4.12	5.46	3.89	5.18	4.10	3.99	4.21	4.17	4.04	0.41-1.56	4.37	0.60
		O1	3.57	3.57	3.89	3.74	3.63	3.62	3.72	3.60	3.75	4.22	3.72	3.61	3.59	3.59	3.84	3.55	3.75	0.20-0.63	3.70	0.17
	O	C31	5.97	6.30	4.32	3.99	4.31	4.25	4.10	4.38	4.20	5.92	4.03	5.44	4.55	4.25	4.29	4.61	4.22	0.54-2.10	4.66	0.75
		O3	5.10	5.23	4.29	4.03	4.09	4.02	4.02	4.17	4.22	5.68	4.03	4.93	4.12	3.97	4.28	4.14	4.14	0.42-1.72	4.38	0.52
Val55	C^{α}	O1	3.72	3.67	3.39	3.36	3.30	3.32	3.34	3.31	3.35	3.54	3.36	3.55	3.32	3.30	3.39	3.34	3.35	0.16-0.39	3.41	0.13
	C^{β}	O1	4.67	4.47	3.69	3.57	3.59	3.66	3.63	3.58	3.61	3.89	3.58	4.34	3.64	3.63	3.67	3.70	3.64	0.21-0.68	3.80	0.35
	$C^{\gamma 1}$	O1	5.31	5.10	3.86	3.86	3.96	4.04	3.93	4.01	3.88	3.87	3.91	4.85	4.05	4.01	3.89	4.14	3.93	0.30-0.80	4.15	0.46
		S4	4.08	3.97	4.54	4.47	4.33	4.45	4.45	4.31	4.44	4.68	4.41	4.37	4.26	4.38	4.48	4.26	4.40	0.29-0.68	4.37	0.17
Ile56	N	O1	3.45	3.29	3.66	3.37	3.23	3.24	3.36	3.17	3.42	4.16	3.34	3.49	3.18	3.20	3.57	3.11	3.40	0.25-0.91	3.39	0.25
	$C^{\gamma 2}$	O2	5.59	5.42	5.03	3.77	3.78	3.78	3.83	3.77	3.91	5.78	3.90	5.13	3.95	3.78	3.91	3.80	3.97	0.45-2.22	4.30	0.74
		O1	3.54	3.43	4.27	3.89	3.79	3.75	3.88	3.72	4.00	4.94	3.83	3.86	3.71	3.74	4.18	3.62	3.93	0.26-1.22	3.89	0.34
Trp59	$C^{\zeta 3}$	C3	5.86	5.55	4.13	4.03	4.16	4.17	4.18	4.06	4.05	4.33	4.05	5.06	4.25	4.19	4.22	4.25	4.12	0.31-0.92	4.39	0.55
		S4	4.44	4.52	4.34	4.05	4.29	4.33	4.27	4.18	4.23	4.65	4.10	4.21	4.26	4.31	4.40	4.10	4.29	0.38-0.68	4.29	0.15
Tyr82	$C^{\epsilon 1}$	O4	5.91	5.95	4.61	4.07	4.17	4.11	4.13	4.04	4.14	4.36	4.18	4.83	4.20	4.08	4.24	4.20	4.27	0.45-0.99	4.44	0.60
		C18	7.15	6.38	3.70	4.00	3.78	3.79	5.07	4.03	3.77	3.75	4.00	5.46	6.01	3.95	3.94	4.94	3.90	0.29-1.71	4.57	1.08

FKBP12 (Atom pair)		Ligand	MD1	MD2	MD3	MD4	MD5	MD6	MD7	MD8	MD9	MD10	MD11	MD12	MD13	MD14	MD15	MD16	MD17	fluctuation range	MD	Std dev.
		C19	7.88	6.98	3.90	4.64	4.27	4.30	5.56	4.69	4.27	4.16	4.56	6.20	6.46	4.53	4.47	5.64	4.38	0.43-1.89	5.11	1.15
	$C^{\epsilon 2}$	O2	4.08	3.67	3.83	3.66	3.57	3.59	3.58	3.52	3.68	4.81	3.62	4.41	3.52	3.57	3.61	3.50	3.60	0.26-1.28	3.75	0.36
	C^{ζ}	O2	4.90	4.47	3.87	3.68	3.65	3.67	3.63	3.60	3.69	4.83	3.68	4.23	3.61	3.63	3.66	3.58	3.65	0.19-1.25	3.88	0.44
		C18	6.19	5.33	3.73	4.05	3.83	3.89	5.03	4.07	3.81	3.89	4.06	5.44	5.72	3.97	3.96	4.74	3.96	0.26-1.59	4.45	0.79
		C19	6.96	6.02	3.76	4.54	4.15	4.24	5.38	4.59	4.13	4.14	4.49	6.02	6.07	4.39	4.34	5.28	4.30	0.40-1.79	4.87	0.91
Tyr82	O^{η}	O2	4.90	4.54	3.12	2.88	2.89	2.91	2.87	2.86	2.89	3.97	2.90	3.26	2.85	2.87	2.88	2.88	2.90	0.17-1.16	3.20	0.64
		C30	5.76	5.35	3.58	3.71	3.72	3.75	3.69	3.73	3.67	3.98	3.69	4.06	3.71	3.72	3.72	3.76	3.70	0.17-0.74	3.96	0.62
		C15	6.05	5.74	4.05	4.05	4.07	4.08	4.03	4.09	3.99	4.19	4.04	4.46	4.12	4.08	4.08	4.16	4.06	0.20-0.68	4.31	0.61
		N1	5.10	4.91	3.40	3.35	3.33	3.33	3.32	3.37	3.29	3.52	3.36	3.83	3.42	3.35	3.38	3.46	3.35	0.23-0.68	3.59	0.55
		C1	4.36	4.42	3.36	3.42	3.36	3.42	3.39	3.44	3.36	3.49	3.44	3.52	3.43	3.40	3.42	3.48	3.40	0.19-0.49	3.54	0.33
		O1	4.59	4.77	3.82	3.98	3.92	4.02	3.96	4.03	3.93	3.95	4.00	3.71	3.97	3.99	3.98	4.05	3.95	0.27-0.57	4.04	0.26
		C2	3.73	3.91	3.47	3.52	3.41	3.45	3.48	3.48	3.44	3.59	3.54	3.63	3.46	3.45	3.48	3.48	3.48	0.17-0.46	3.53	0.13
		C3	3.83	4.33	4.69	4.76	4.66	4.72	4.74	4.75	4.66	4.72	4.79	4.31	4.68	4.72	4.69	4.63	4.73	0.22-0.73	4.61	0.24
		N7	4.72	4.75	4.05	4.06	3.94	3.94	4.00	3.97	4.00	4.25	4.08	4.58	4.01	3.95	4.06	4.06	4.01	0.26-0.71	4.14	0.27
		O4	3.73	3.69	4.31	3.96	3.98	3.94	3.97	3.88	4.00	4.28	4.00	4.60	3.88	3.90	3.97	3.80	4.04	0.39-0.86	4.00	0.22
		C17	5.13	4.93	3.67	4.06	3.72	3.73	3.95	3.97	3.79	4.09	4.01	4.97	3.93	3.83	3.94	4.08	3.88	0.27-1.25	4.10	0.46
		C18	5.05	4.31	3.45	3.47	3.35	3.39	4.30	3.46	3.40	3.60	3.46	4.65	4.69	3.42	3.44	3.93	3.46	0.24-1.38	3.81	0.56
		C19	5.94	5.20	3.75	4.09	3.84	3.89	4.79	4.10	3.89	4.07	4.03	5.27	5.14	3.98	4.00	4.56	3.98	0.33-1.49	4.39	0.65
		C22	6.00	6.21	4.15	5.07	4.48	4.49	4.02	4.96	4.57	4.90	4.95	5.82	3.59	4.67	4.85	4.73	4.69	0.45-1.38	4.83	0.68
His87	C^{β}	O4	4.95	4.94	4.30	3.75	4.22	4.28	4.09	4.02	3.90	3.95	4.09	4.13	4.08	4.04	3.89	4.29	4.07	0.38-0.86	4.18	0.33
		C17	5.09	4.83	4.66	3.94	4.84	4.70	4.32	4.36	4.31	4.00	4.28	5.38	5.12	4.58	4.27	5.21	4.51	0.37-0.78	4.61	0.42
His87	C^{β}	C18	4.43	4.00	4.28	3.76	4.52	4.33	4.57	4.06	4.01	3.82	4.01	5.09	5.96	4.24	3.99	5.10	4.16	0.34-1.00	4.37	0.56
		C19	4.57	4.02	4.99	4.40	5.27	5.03	5.12	4.70	4.76	4.45	4.59	5.94	6.68	4.98	4.70	5.86	4.85	0.45-1.06	4.99	0.65
	C^{γ}	O4	5.84	5.14	4.51	4.40	4.35	4.47	4.39	4.55	4.46	4.59	4.53	4.05	4.04	4.34	4.36	4.31	4.31	0.39-1.01	4.51	0.42
		O5	7.40	6.43	4.16	4.15	4.23	4.30	4.31	4.47	4.15	4.01	4.38	5.13	4.30	4.35	4.20	4.89	4.23	0.43-0.98	4.65	0.91
		C17	5.36	4.87	4.12	4.40	4.17	4.05	4.19	4.47	4.54	4.47	4.27	4.65	4.32	4.29	4.42	4.49	4.15	0.34-0.80	4.43	0.32
		C18	4.60	4.31	3.91	4.58	3.95	3.77	4.35	4.43	4.51	4.63	4.26	4.29	4.98	4.10	4.43	4.34	4.02	0.37-0.92	4.32	0.30
	$N^{\delta 1}$	S8	6.91	6.14	4.19	4.94	4.14	3.95	4.43	4.86	4.91	4.94	4.65	4.72	4.16	4.45	4.81	4.57	4.31	0.35-1.26	4.77	0.74
		O4	6.53	5.67	4.50	4.96	4.31	4.30	4.52	4.90	4.89	5.07	4.77	4.31	4.06	4.52	4.77	4.35	4.44	0.46-1.31	4.76	0.60
		O5	7.97	6.76	3.74	4.46	3.86	3.49	4.09	4.50	4.39	4.28	4.27	5.03	4.09	4.14	4.40	4.73	3.95	0.43-1.44	4.60	1.12
		C17	5.82	5.35	3.91	4.94	3.78	3.64	4.24	4.66	5.01	5.04	4.44	4.47	3.91	4.21	4.81	4.19	4.10	0.32-1.07	4.50	0.60

Atom pair																					
FKBP12		Ligand	MD1	MD2	MD3	MD4	MD5	MD6	MD7	MD8	MD9	MD10	MD11	MD12	MD13	MD14	MD15	MD16	MD17	fluctuation range	MD	Std dev.
		C18	5.03	4.89	3.96	5.23	3.74	3.79	4.53	4.76	5.09	5.33	4.62	4.17	4.51	4.22	4.92	4.16	4.22	0.40-1.08	4.54	0.50
		C22	6.11	5.68	4.39	5.25	4.31	4.06	4.52	5.03	5.60	5.39	4.74	5.18	3.86	4.73	5.31	4.59	4.55	0.44-1.29	4.90	0.61
	$C^{\delta 2}$	C18	4.90	4.60	4.12	5.20	4.21	3.91	4.57	4.95	4.93	5.20	4.69	4.19	4.87	4.44	4.89	4.33	4.31	0.57-1.13	4.61	0.39
		C19	4.47	4.61	4.32	5.75	4.50	3.93	4.87	5.33	5.45	5.70	4.95	4.66	5.31	4.78	5.41	4.60	4.61	0.64-1.28	4.90	0.51
	$C^{\epsilon 1}$	O5	8.65	6.90	4.12	4.84	4.14	4.04	4.46	4.92	4.76	4.72	4.68	5.35	4.23	4.55	4.73	5.07	4.26	0.51-1.45	4.97	1.16
		C17	6.36	5.75	4.04	5.64	3.82	3.74	4.64	5.19	5.51	5.72	4.88	4.36	3.79	4.49	5.30	4.13	4.34	0.32-1.25	4.81	0.81
His87	$C^{\epsilon 1}$	C18	5.55	5.49	4.21	6.11	3.91	3.92	4.84	5.43	5.75	6.17	5.20	3.99	4.07	4.59	5.57	4.05	4.60	0.39-1.35	4.91	0.80
		C21	5.88	5.77	4.40	6.27	4.17	3.95	5.23	5.63	6.38	6.44	5.22	5.04	4.13	4.99	6.04	4.35	4.79	0.49-1.63	5.22	0.83
		C22	6.49	5.86	4.12	5.73	3.95	3.74	4.84	5.29	5.84	5.86	4.88	4.84	3.81	4.69	5.54	4.25	4.43	0.40-1.43	4.95	0.84
	$N^{\epsilon 2}$	C18	5.52	5.38	4.34	6.14	4.20	4.02	4.89	5.57	5.71	6.15	5.28	4.01	4.30	4.75	5.61	4.16	4.69	0.45-1.17	4.98	0.74
		C19	4.82	5.32	4.40	6.66	4.31	3.88	5.14	5.85	6.19	6.65	5.46	4.21	4.55	4.93	6.06	4.15	4.88	0.50-1.49	5.14	0.88
		C20	5.01	5.44	4.45	6.71	4.40	3.92	5.35	5.94	6.41	6.72	5.44	4.80	4.63	5.11	6.22	4.35	4.93	0.53-1.70	5.28	0.86
		C21	5.79	5.58	4.43	6.23	4.39	4.09	5.33	5.73	6.16	6.30	5.20	5.14	4.45	5.11	5.93	4.51	4.79	0.54-1.59	5.24	0.71
Ile90	C^{β}	O4	4.71	4.75	3.81	4.25	4.03	4.01	4.16	4.14	4.06	3.98	4.20	4.24	4.30	4.11	4.03	4.27	4.02	0.28-0.55	4.18	0.24
	C^{δ}	O5	3.97	4.20	3.72	3.74	3.63	3.73	3.78	3.72	3.67	3.68	3.73	3.77	3.70	3.75	3.68	3.81	3.66	0.38-0.72	3.76	0.14
	$C^{\gamma 2}$	O4	5.77	5.78	3.73	4.36	4.04	3.67	4.12	3.99	4.07	3.87	4.14	4.06	4.37	3.93	3.96	3.89	3.87	0.36-0.69	4.21	0.62
Ile91	C^{δ}	C3	4.95	4.74	3.71	3.71	3.75	3.73	3.72	3.73	3.74	3.91	3.72	4.65	3.79	3.75	3.78	3.97	3.74	0.23-0.70	3.95	0.41
	$C^{\gamma 1}$	C3	3.89	3.87	4.44	4.36	4.53	4.37	4.37	4.47	4.55	4.64	4.41	4.14	4.55	4.48	4.46	4.65	4.44	0.28-0.61	4.39	0.22
